# Dietary amino acid patterns and epilepsy among the PERSIAN cohort population: A cross‐sectional study

**DOI:** 10.1113/EP093525

**Published:** 2026-10-02

**Authors:** Hamid Abbasi, Ali Jafari, Saeid Doaei, Akram Kooshki, Raul Zamora‐Ros

**Affiliations:** ^1^ Student Research Committee, Department of Clinical Nutrition and Dietetics Faculty of Nutrition Sciences and Food Technology National Nutrition and Food Technology Research Institute Shahid Beheshti University of Medical Sciences Tehran Iran; ^2^ Student Research Committee, Department of Community Nutrition Faculty of Nutrition Sciences and Food Technology National Nutrition and Food Technology Research Institute Shahid Beheshti University of Medical Sciences Tehran Iran; ^3^ Systematic Review and Meta‐analysis Expert Group (SRMEG) Universal Scientific Education and Research Network (USERN) Tehran Iran; ^4^ Department of Community Nutrition Faculty of Nutrition and Food Technology National Nutrition and Food Technology Research Institute Shahid Beheshti University of Medical Sciences Tehran Iran; ^5^ Unit of Nutrition and Cancer, Cancer Epidemiology Research Program, Catalan Institute of Oncology (ICO), Bellvitge Biomedical Research Institute (IDIBELL) L'Hospitalet deLlobregat Barcelona Spain; ^6^ Non‐Communicable Diseases Research Center Department of Nutrition and Biochemistry School of Medicine Sabzevar University of Medical Sciences Sabzevar Iran

**Keywords:** amino acid, diet, dietary patterns, epilepsy, Persian cohort, protein intake

## Abstract

Epilepsy is characterized by the occurrence of at least two unprovoked seizures occurring more than 24 h apart, reflecting a predisposition to recurrent epileptic events. The role of dietary amino acids (AAs) in epilepsy is not clearly understood. To the best of our knowledge, this study is the first to investigate the link between the odds of developing epilepsy and dietary AA patterns. This cross‐sectional study, derived from the Prospective Epidemiologic Research Studies in Iran (PERSIAN) Sabzevar Cohort Study, included 112 individuals diagnosed with prevalent epilepsy and 3951 participants without epilepsy. Dietary intake was estimated using a validated semi‐quantitative food frequency questionnaire. Principal component analysis was applied to identify two distinct dietary AA patterns: one predominantly derived from animal sources and the other from plant food sources. Participants with epilepsy were more likely to be male, have lower levels of education, be married, be unemployed and also to exhibit lower intakes of all assessed AAs. In the fully adjusted model, no significant association was observed between the animal AA pattern and the odds of having epilepsy (odds ratio (OR) (Q4 vs. Q1): 0.62, 95% CI: 0.35–1.08, *P *= 0.094), although the *P*‐value for trend across quartiles was statistically significant (*P*_trend _= 0.002). By contrast, higher adherence to the plant AA pattern was associated with 63% lower odds of having epilepsy (OR (Q4 vs. Q1): 0.37, 95% CI: 0.20–0.67, *P *= 0.001). The *P*‐value for trend across quartiles was significant (*P*_trend _= 0.001). In conclusion, greater adherence to the plant‐based AA pattern was associated with lower odds of having epilepsy. These findings may inform dietary strategies, but prospective studies are needed to determine whether these associations reflect a causal relationship.

## Abstract (highlights)

- **What is the central question of this study?**
  Is there an association between dietary amino acid patterns and the odds of having epilepsy among the PERSIAN cohort population.
- **What is the main finding and its importance?**
  Higher adherence to a plant‐based amino acid pattern is associated with a 63% reduction in the odds of having epilepsy. This suggests that dietary amino acid choices may influence epilepsy odds, which highlights the potential for dietary interventions in preventing the condition.

## 1 INTRODUCTION

Epilepsy, ranked as the third most prevalent neurological disorder, is characterized by the occurrence of at least two unprovoked seizures or reflex seizures separated by more than 24 h (Devinsky et al., 2018; Fisher et al., 2014). Worldwide trends show a rising prevalence and incidence of epilepsy, affecting more than 70 million individuals (Beghi, 2020; Pong et al., 2023), and resulting in 125,000 deaths associated with epilepsy annually (Ghosh et al., 2021; Mishra et al., 2021). Various subtypes are considered for epileptic seizures; however, it is primarily categorized into four major classes: focal, generalized, unknown and unclassified (Beniczky et al., 2025). Several clinical manifestations may accompany epilepsy, most importantly movement disorders, changes in consciousness levels and frequent seizures (Fisher and Bonner, 2018). Regarding the high prevalence of epilepsy and its related health outcomes, epilepsy management is considered to be a key priority. The aetiopathogenesis of epilepsy is complex and multifactorial, involving numerous structural, immune, genetic, metabolic, infectious and other still unknown factors (World Health Organization, 2024).

Amino acids (AAs) serve as the fundamental monomers of proteins and act as nitrogenous precursors for bioactive molecules like neurotransmitters and hormones (Lopez and Mohiuddin, 2024; Dietzen, 2018). A few publications have suggested that some AAs may have a therapeutic role in the management of epilepsy (Gruenbaum et al., 2019; Gietzen et al., 1996; Evangeliou et al., 2009). A systematic literature review on clinical and animal studies demonstrated paradoxical effects of a mixture of branched‐chain AAs (BCAAs; i.e., isoleucine, valine and leucine) on seizure activity, likely arising from their intricate interactions with glucose, glutamate, glutamine and ammonia metabolism, as well as the mechanistic target of rapamycin (mTOR) signalling pathway and neurotransmitter synthesis (Gruenbaum et al., 2019). A pilot study found that using BCAAs along with a ketogenic diet resulted in seizure reduction in individuals diagnosed with various types of epilepsy (Evangeliou et al., 2009). Additionally, another study indicated that certain dietary AAs such as threonine, isoleucine and histidine may exhibit therapeutic potential in mitigating the exacerbation of epilepsy in affected rat models (Gietzen et al., 1996).

Despite the burgeoning corpus of evidence elucidating the link between dietary AAs and several non‐neurological chronic diseases such as type 2 diabetes, hypertension, chronic kidney disease and metabolic markers (Yu et al., 2021; Zheng et al., 2016; Teymoori et al., 2017; Asghari et al., 2022), the associations between AAs and the odds of having epilepsy have not been explored yet. Thus, the aim of this cross‐sectional research is to evaluate whether certain dietary patterns of AAs are associated with a lower prevalence of epilepsy among adults in the Prospective Epidemiologic Research Studies in Iran (PERSIAN) cohort.

## 2 METHODS

### 2.1 Ethical approval

This study was approved by the Research Ethics Committee at Sabzevar University of Medical Sciences in Sabzevar, Iran (approval code: IR.MEDSAB.REC.1403.158). All procedures involving human participants were conducted in accordance with the *Declaration of Helsinki* and its subsequent amendments, as well as the ethical guidelines set forth by relevant national and institutional research councils. Written informed consent was obtained from all participants prior to their involvement in the study. The research was conducted in compliance with ethical standards, ensuring the rights and welfare of the subjects were prioritized throughout the study.

### 2.2 Study design

In this cross‐sectional research, we used baseline data from a subset of the Prospective Epidemiologic Research Studies in Iran (PERSIAN) cohort study in Sabzevar city. The PERSIAN cohort is a forward‐looking, longitudinal research initiative targeting a representative sample of the Iranian adult population. This large cohort includes roughly 180,000 individuals aged 35–70 from 19 diverse regions across Iran (Poustchi et al., 2018; Eghtesad et al., 2017; Ahmadi et al., 2021). Its primary objective is to investigate the prevalence and incidence of non‐communicable diseases and to examine their associated risk factors, while accounting for the influence of cultural practices, lifestyle behaviours and environmental factors specific to this population (Poustchi et al., 2018; Eghtesad et al., 2017). Specifically, the Sabzevar cohort study is a subset of the PERSIAN cohort, based in the northeastern region of Iran, particularly Tawheed town in Sabzevar city (Ahmadi et al., 2021; Bakhshimoghaddam et al., 2024). Tawheed town, a part of Sabzevar, has a total population of 21,973, with 10,790 men and 11,183 women. This area was chosen due to its low immigration rate and diverse ethnic and racial composition, facilitating long‐term follow‐up. Using sequential sampling, 4218 individuals who were enrolled at the PERSIAN cohort centre were considered for the present study.

### 2.3 Study population and data collection

Of the 4218 participants enrolled from the Sabzevar‐PERSIAN cohort, a total of 155 were excluded, due to missing AA (*n* = 94), demographic (*n* = 2) or clinical (*n* = 42) data, being pregnant or lactating (*n* = 7), or unrealistic reporting energy intakes (<1000 or >5500 kcal/day) (*n* = 10) (Figure 1). Ultimately, a total of 4063 participants (112 epileptic cases and 3951 non‐cases) were included in this study. At baseline, diagnosis of prevalent epilepsy was established through a specialist physician. All data was collected at baseline from January 2017 to May 2020.

**FIGURE 1:**
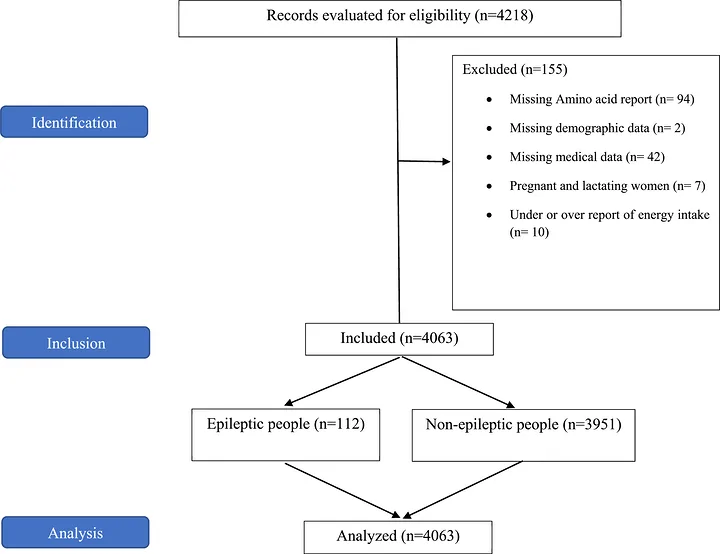
Study flowchart according to the Strengthening the Reporting of Observational Studies in Epidemiology (STROBE) guidelines.

### 2.4 Sociodemographic and lifestyle characteristics

A standardized questionnaire was employed to collect comprehensive data encompassing demographic variables, socioeconomic status, individual and familial medical histories including chronic pathologies, reproductive history, smoking and alcohol consumption (Poustchi et al., 2018). The questionnaires provided information on recent and historical pharmacological interventions and reproductive status, and also included detailed documentation of nutritional supplement intake.

The anthropometric measurements of all participants were conducted while they wore lightweight clothes and remained barefoot. Body weight was quantified in kilograms, rounded to the nearest 0.1 kg, employing a calibrated mechanical scale (Seca 755, Hamburg, Germany). Height was measured in centimetres (cm), also rounded to the nearest 0.1 cm, utilizing a precision stadiometer (Seca 204). The body mass index (BMI) for each participant was calculated by dividing the weight in kilograms by the square of height in metres.

To assess physical activity levels, the Interventional Physical Activity Questionnaire (IPAQ) short form was utilized, which has been previously validated within the Iranian population (Moghaddam et al., 2012). Physical activity was stratified into four distinct categories: level 1: sedentary lifestyle; level 2: occasional walking or standing; level 3: mainly non‐outdoor activities; level 4: non‐indoor activities resulting in an enhancement in heart rate and sweating. According to gathered data, we quantified physical activity expenditure in terms of metabolic equivalent task hour per day (MET‐h/day). Furthermore, principal component analysis (PCA) was employed to derive a composite socioeconomic status (SES) variable, integrating metrics related to employment status, household amenities, and participants’ educational attainment (Vyas and Kumaranayake, 2006).

### 2.5 Dietary assessment

Habitual dietary intake data of all participants were gathered through a validated semi‐quantitative food frequency questionnaire (FFQ), containing 115 food items highly consumed in the Iranian diet (Malekshah et al., 2006). The frequency of food consumed over the preceding year was estimated via a face‐to‐face interview. The Iranian food composition table (FCT) was used to ascertain the energy and nutrient intakes and analysis was performed using Nutritionist 4 software (First Databank Division, the Hearst Corporation, New York City, New York, USA), which specifically accommodated Iranian food items. AA profiles were calculated, including both essential (histidine, isoleucine, leucine, lysine, methionine, phenylalanine, threonine, tryptophan and valine) and non‐essential/conditionally essential AAs (alanine, asparagine, aspartic acid, glutamic acid, serine, glycine, proline, cysteine, tyrosine, glutamine and arginine), allowing for a comprehensive assessment of dietary AA intake patterns.

### 2.6 Covariates

A set of potential confounders was identified a priori based on prior evidence linking them to epilepsy odds, dietary AA intake or both, and was included in the fully adjusted models. Age (years) and sex (male/female) were recorded at baseline (Ghebrehiwet et al., 2023). Educational attainment was categorized as illiterate, elementary school, middle school, high school diploma, associate degree, bachelor's degree, master's degree or doctor of philosophy, and was used as a proxy for socioeconomic position (Ghebrehiwet et al., 2023). Marital status (single, married, widowed, divorced) and employment status (employed vs. unemployed) were self‐reported and served as additional socioeconomic indicators (Tedrus et al., 2015). BMI was calculated as weight in kilograms divided by height in metres squared and was treated as a continuous variable to account for differences in adiposity (Li et al., 2025). Prevalent diabetes and hypertension were determined through a physician diagnosis (Doherty et al., 2022), while any prevalent cancer was identified from self‐reported medical history (Pedersen et al., 2026). All covariates were assessed at the time of enrolment using identical standardized procedures for cases and non‐cases, and they were entered simultaneously into the fully adjusted logistic regression models to estimate the independent association between dietary amino acid patterns and the odds of having epilepsy.

### 2.7 Statistical analysis

AA pattern extraction was based on the correlation matrix, and components with an eigenvalue above 0.25 were considered dominant (Figure 2). The correlations among AAs and sampling adequacy were evaluated using Bartlett's test of sphericity and the Kaiser–Meyer–Olkin test, respectively. The distribution normality of all variables was verified using the Kolmogorov–Smirnov test and histogram analysis. Quantitative variables are presented as means ± standard deviation, while qualitative variables are expressed as percentages. The trends of categorical and continuous variables across quartiles of each dietary AA pattern score (median value) were assessed using the chi‐squared test and analysis of variance (ANOVA) for the study characteristics table, and the chi‐squared and analysis of covariance (ANCOVA) for nutrient characteristic table. We cross‐sectionally analysed the association between quartiles of two extracted AA patterns and epilepsy odds through logistic regression models, with results expressed as odds ratio (OR) and 95% confidence interval (CI). The *P*‐value for trend was estimated by assigning scores of each dietary AA pattern quartile as a continuous variable. The regression models were adjusted for potential confounders such as sex, age, education level, BMI, prevalent diabetes, prevalent hypertension, marital status, job and any prevalent cancer. *Post hoc* pairwise comparisons between quartiles were performed using Tukey's honestly significant difference (HSD) test for continuous variables and Bonferroni‐adjusted proportion tests for categorical variables. Statistical analyses were executed using RStudio version 2026.01.0 in conjunction with R version 4.5.2. A *P*‐value of ≤ 0.05 was considered statistically significant.

**FIGURE 2:**
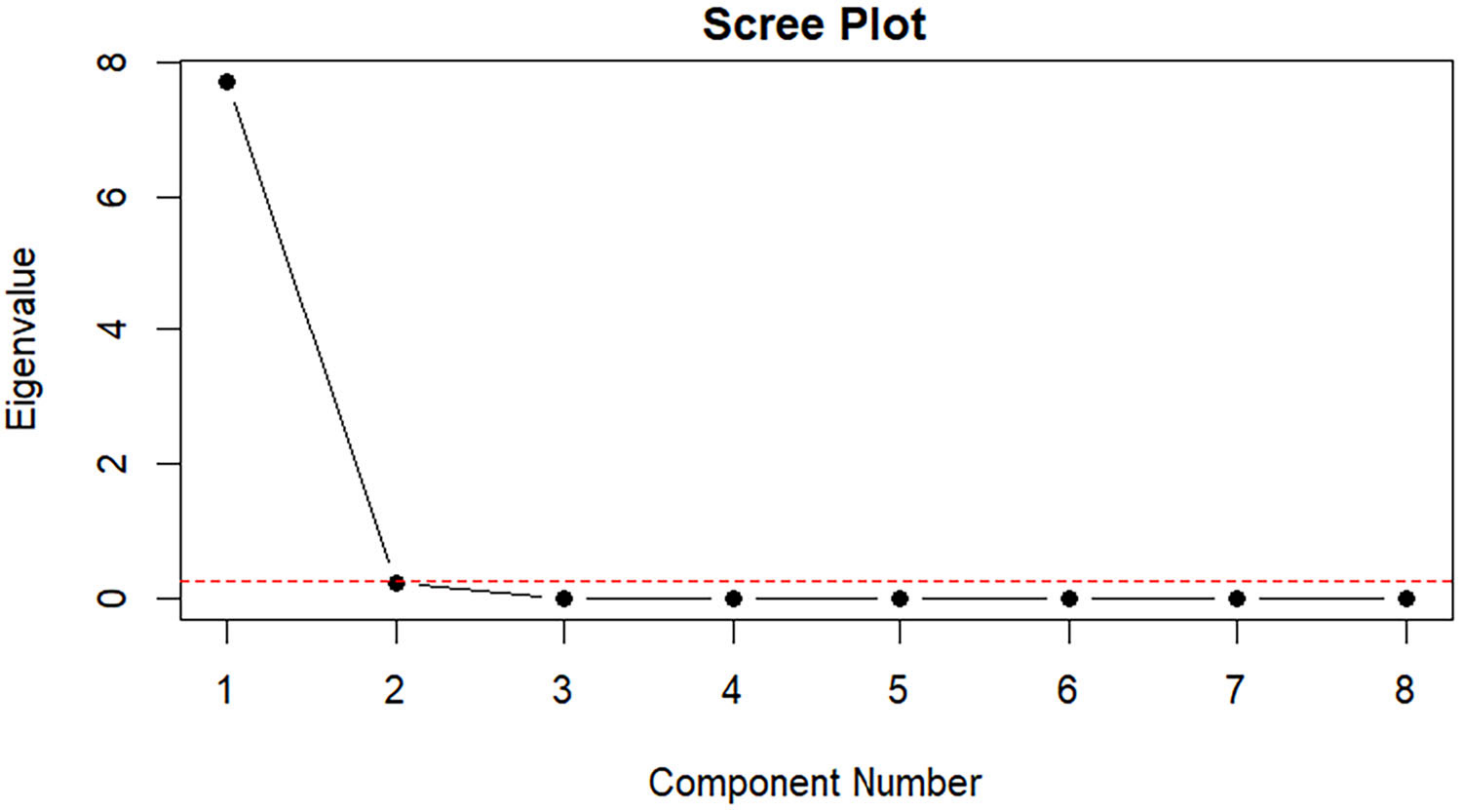
Scree plot for principal component analysis to identify dietary amino acid patterns. Eight groups of amino acids, based on their percentage contribution to the total protein, were utilized as input variables. Factors with eigenvalues greater than 0.25 were identified as part of the dietary amino acid pattern.

## 3 RESULTS

PCA was employed with varimax rotation to identify dietary AA patterns, which are presented in Table 1. Notably, the dietary AA patterns identified in this study included Pattern 1, which was predominantly associated with animal‐based foods, and Pattern 2, which was primarily related to plant‐based foods. Thus, the animal AA pattern (AAAP) and the plant AA pattern (PAAP) were used to refer to Pattern 1 and Pattern 2, respectively. As illustrated in Table 2, the mean age was 48.9 ± 9.1 years in individuals with epilepsy and 49.3 ± 8.8 years in non‐cases. Significant differences across quartiles of both AAAP and PAAP were observed for age (*P *< 0.001), sex distribution (male proportion increasing from 31.1% in Q1 to 60.5% in Q4 for AAAP, and from 23.2% to 73.2% for PAAP, *P *< 0.001) and educational attainment. Marital status also varied, with married participants predominating in higher quartiles (*P *< 0.001). Clinical variables showed significant associations: hypertension prevalence decreased across quartiles of both patterns (28.3% in Q1 to 19.3% in Q4 for AAAP, *P *< 0.001; 25.6% to 19.2% for PAAP, *P* = 0.003), while diabetes was more frequent among epilepsy cases (23.2% vs. 13.8%, *P* = 0.042). Significant pairwise differences across quartiles of the AA patterns for demographic and clinical characteristics are presented in Supporting information Tables S1 and S2.

**TABLE 1:** Factor‐loading matrix for two principal dietary amino acid patterns determined through principal component analysis.

	Dietary amino acid pattern	
Amino acid	Animal amino‐acid pattern	Plant amino‐acid pattern
Lysine	0.924	—
Aspartic acid	0.879	—
Arginine	0.830	—
Alanine	0.803	—
Threonine	0.803	—
Histidine	0.776	—
Methionine	0.772	—
Valine	0.755	—
Isoleucine	0.755	—
Glycine	0.752	—
Tyrosine	0.750	—
Leucine	0.752	—
Proline	—	0.894
Glutamic acid	—	0.892
Cysteine	—	0.864
Phenylalanine	—	0.752
Serine	—	0.738
Tryptophan	—	0.728
Cumulative variance explained (%)^1^	53.23	45.65

Only absolute values ≥0.30 are displayed to simplify interpretation. ^1^The patterns explain the variance percentage in the total intake of amino acid groups. Kaiser–Meyer–Olkin test: 0.929; *P* ≤ 001.

**TABLE 2:** Study characteristics of participants stratified by quartiles of the dietary amino acid patterns.

	Epilepsy			Animal amino‐acid pattern					Plant amino‐acid pattern				
	No	Yes		Q1	Q2	Q3	Q4		Q1	Q2	Q3	Q4	
Characteristic	(*n* = 3951)^1^	(*n* = 112)^1^	*P* ^2^	(*n* = 1016)^1^	(*n* = 1016)^1^	(*n* = 1016)^1^	(*n* = 1015)^1^	*P* ^3^	(*n* = 1016)^1^	(*n* = 1016)^1^	(*n* = 1016)^1^	(*n* = 1015)^1^	*P* ^3^
**Age (years)**	49.29 ± 8.75	48.91 ± 9.13	0.655	50.93 ± 9.07	49.88 ± 8.59	48.80 ± 8.70	47.48 ± 8.27	<0.001	49.63 ± 8.68	49.40 ± 8.81	49.83 ± 8.62	48.24 ± 8.84	<0.001
**Males**	1746 (44.19)	53 (47.32)	0.511	316 (31.10)	410 (40.35)	459 (45.18)	614 (60.49)	<0.001	236 (23.23)	317 (31.20)	503 (49.51)	743 (73.20)	<0.001
**Body mass index**			0.803					0.035					0.109
Normal	897 (22.70)	24 (21.43)		256 (25.20)	207 (20.37)	218 (21.46)	240 (23.65)		212 (20.87)	225 (22.15)	232 (22.83)	252 (24.83)	
Obese	1227 (31.06)	39 (34.82)		314 (30.91)	316 (31.10)	322 (31.69)	314 (30.94)		340 (33.46)	331 (32.58)	317 (31.20)	278 (27.39)	
Overweight	1776 (44.95)	47 (41.96)		427 (42.03)	475 (46.75)	469 (46.16)	452 (44.53)		451 (44.39)	442 (43.50)	456 (44.88)	474 (46.70)	
Underweight	51 (1.29)	2 (1.79)		19 (1.87)	18 (1.77)	7 (0.69)	9 (0.89)		13 (1.28)	18 (1.77)	11 (1.08)	11 (1.08)	
**Education**			0.972					<0.001					<0.001
Below high school diploma	2524 (63.88)	74 (66.07)		641 (63.09)	684 (67.31)	634 (62.40)	639 (62.97)		654 (64.37)	656 (64.56)	636 (62.60)	652 (64.24)	
Associate degree	254 (6.43)	7 (6.25)		40 (3.94)	50 (4.92)	90 (8.86)	81 (7.98)		55 (5.41)	61 (6.00)	81 (7.97)	64 (6.31)	
Bachelor's degree	509 (12.88)	11 (9.82)		59 (5.81)	120 (11.81)	158 (15.55)	183 (18.03)		115 (11.32)	126 (12.40)	133 (13.09)	146 (14.38)	
Postgraduate education	137 (3.47)	4 (3.57)		15 (1.48)	30 (2.95)	42 (4.13)	54 (5.32)		27 (2.66)	36 (3.54)	41 (4.03)	37 (3.64)	
Illiterate	527 (13.34)	16 (14.29)		261 (25.69)	132 (12.99)	92 (9.06)	58 (5.71)		165 (16.24)	137 (13.48)	125 (12.30)	116 (11.43)	
**Marital status**			0.088					<0.001					<0.001
Single	22 (0.56)	2 (1.79)		6 (0.59)	8 (0.79)	3 (0.30)	7 (0.69)		8 (0.79)	10 (0.98)	3 (0.30)	3 (0.30)	
Married	3688 (93.34)	108 (96.43)		908 (89.37)	952 (93.70)	969 (95.37)	967 (95.27)		919 (90.45)	944 (92.91)	952 (93.70)	981 (96.65)	
Widowed	186 (4.71)	1 (0.89)		85 (8.37)	43 (4.23)	31 (3.05)	28 (2.76)		67 (6.59)	50 (4.92)	48 (4.72)	22 (2.17)	
Divorced	55 (1.39)	1 (0.89)		17 (1.67)	13 (1.28)	13 (1.28)	13 (1.28)		22 (2.17)	12 (1.18)	13 (1.28)	9 (0.89)	
**Having a job**	1676 (42.42)	44 (39.29)	0.952	319 (31.40)	370 (36.42)	450 (44.29)	581 (57.24)	<0.001	295 (29.04)	330 (32.48)	439 (43.21)	656 (64.63)	<0.001
**Stroke**	46 (1.16)	6 (5.36)	<0.001	21 (2.07)	10 (0.98)	14 (1.38)	7 (0.69)	0.036	13 (1.28)	22 (2.17)	7 (0.69)	10 (0.99)	0.020
**Diabetes**	544 (13.77)	26 (23.21)	0.005	165 (16.24)	143 (14.07)	142 (13.98)	120 (11.82)	0.042	142 (13.98)	135 (13.29)	148 (14.57)	145 (14.29)	0.858
**Hypertension**	921 (23.31)	28 (25.00)	0.677	287 (28.25)	236 (23.23)	230 (22.64)	196 (19.31)	<0.001	260 (25.59)	252 (24.80)	242 (23.82)	195 (19.21)	0.003
**Any cancer**	36 (0.91)	5 (4.46)	<0.001	10 (0.98)	13 (1.28)	9 (0.89)	9 (0.89)	0.787	17 (1.67)	7 (0.69)	6 (0.59)	11 (1.08)	0.061
**Smoking**	605 (15.31)	21 (18.75)	0.595	118 (11.61)	133 (13.09)	157 (15.45)	218 (21.48)	<0.001	114 (11.22)	122 (12.01)	171 (16.83)	219 (21.58)	<0.001
**Alcohol**	278 (7.04)	5 (4.46)	0.292	40 (3.94)	51 (5.02)	65 (6.40)	127 (12.51)	<0.001	56 (5.51)	65 (6.40)	59 (5.81)	103 (10.15)	<0.001
**MET (kcal/kg h)**	38.87 ± 7.32	37.35 ± 8.47	0.031	38.92 ± 7.42	39.09 ± 7.49	38.45 ± 6.92	38.84 ± 7.58	0.241	38.15 ± 6.10	38.16 ± 5.97	38.54 ± 7.31	40.46 ± 9.30	<0.001

^1^ Data are expressed as means ± SD or *n* (%).

^2^ Chi‐square or Student's *t*‐test was used, as applicable.

^3^ Derived from one‐way analysis of means (ANOVA) test or Pearson's chi‐squared test, as applicable.

Abbreviation: MET: metabolic equivalent of task.

Table 3 presents dietary nutrient intakes of participants with and without epilepsy. Individuals with epilepsy showed lower intake of vitamin D (36.91 ± 20.91 vs. 43.10 ± 27.34, *P *< 0.001). In addition, intake of all assessed AAs increased across quartiles of both patterns (*P *< 0.001). Corresponding nutrient‐level comparisons are provided in Supporting information Table S3.

**TABLE 3:** Nutrient characteristics of participants stratified by quartiles of the dietary amino acid patterns.

	Epilepsy			Animal amino‐acid pattern					Plant amino‐acid pattern				
	No	Yes		Q1	Q2	Q3	Q4		Q1	Q2	Q3	Q4	
Characteristic	(*n* = 3951)^1^	(*n* = 112)^1^	*P* ^2^	(*n* = 1016)^1^	(*n* = 1016)^1^	(*n* = 1016)^1^	(*n* = 1015)^1^	*P* ^3^	(*n* = 1016)^1^	(*n* = 1016)^1^	(*n* = 1016)^1^	(*n* = 1015)^1^	*P* ^3^
**Energy (kcal/day)**	2499.44 ± 784.44	2442.87 ± 839.47	0.453	1971.08 ± 617.86	2320.36 ± 625.67	2605.09 ± 625.25	3095.58 ± 794.03	<0.001	1935.74 ± 619.96	2243.60 ± 542.08	2563.00 ± 575.55	3249.93 ± 715.74	<0.001
**Macronutrients**													
Carbohydrate (g)	412.88 ± 137.89	404.78 ± 149.05	0.541	341.17 ± 118.93	389.53 ± 120.49	430.42 ± 121.49	489.57 ± 145.41	<0.001	304.01 ± 103.30	364.39 ± 85.96	427.25 ± 95.39	555.13 ± 120.93	<0.001
Protein (g)	78.72 ± 26.03	76.32 ± 28.08	0.339	57.99 ± 18.60	71.06 ± 18.13	81.83 ± 17.79	103.75 ± 24.64	<0.001	61.12 ± 21.73	70.78 ± 19.20	80.14 ± 19.16	102.59 ± 23.89	<0.001
Total fat (g)	64.59 ± 25.35	62.99 ± 26.62	0.511	44.77 ± 15.75	57.92 ± 16.93	67.71 ± 19.31	87.83 ± 26.16	<0.001	59.11 ± 24.18	61.28 ± 23.69	64.52 ± 23.66	73.30 ± 27.50	<0.001
**Micronutrients**													
Vitamin A (µg)	714.69 ± 464.77	698.36 ± 439.06	0.713	437.33 ± 194.54	606.99 ± 233.27	745.53 ± 270.57	1067.47 ± 694.79	<0.001	705.32 ± 530.01	689.29 ± 474.57	691.08 ± 368.64	771.34 ± 464.14	<0.001
Vitamin E (mg)	7.20 ± 3.26	7.17 ± 3.20	0.932	4.87 ± 1.82	6.38 ± 2.12	7.57 ± 2.40	9.97 ± 3.88	<0.001	7.04 ± 3.38	6.84 ± 2.97	7.08 ± 3.01	7.83 ± 3.56	<0.001
Vitamin D (IU)	43.10 ± 27.34	36.91 ± 20.91	0.018	24.66 ± 12.14	35.56 ± 15.04	45.33 ± 17.37	66.18 ± 36.78	<0.001	39.41 ± 23.91	40.76 ± 24.42	41.90 ± 24.04	49.63 ± 33.95	<0.001
Vitamin K (µg)	165.84 ± 102.39	160.02 ± 96.55	0.553	112.67 ± 66.37	150.32 ± 78.28	179.74 ± 90.92	220.04 ± 129.76	<0.001	172.21 ± 115.90	157.35 ± 91.79	163.08 ± 97.37	170.09 ± 101.77	>0.9
Vitamin C (mg)	142.13 ± 81.79	135.47 ± 74.54	0.395	93.11 ± 47.37	126.50 ± 60.22	155.00 ± 70.54	193.22 ± 101.89	<0.001	147.02 ± 95.66	134.59 ± 73.34	139.35 ± 74.36	146.82 ± 80.50	0.7
Thiamin (mg)	1.64 ± 0.60	1.60 ± 0.63	0.459	1.34 ± 0.52	1.53 ± 0.52	1.69 ± 0.52	1.99 ± 0.64	<0.001	1.17 ± 0.50	1.42 ± 0.36	1.69 ± 0.36	2.27 ± 0.53	<0.001
Riboflavin (mg)	1.75 ± 0.65	1.69 ± 0.64	0.318	1.22 ± 0.34	1.56 ± 0.35	1.83 ± 0.38	2.40 ± 0.74	<0.001	1.54 ± 0.66	1.62 ± 0.58	1.76 ± 0.56	2.08 ± 0.65	<0.001
Niacin (mg)	18.16 ± 6.51	18.03 ± 7.31	0.842	13.31 ± 4.56	16.25 ± 4.53	18.91 ± 4.60	24.15 ± 6.67	<0.001	14.80 ± 6.62	16.34 ± 5.08	18.28 ± 4.97	23.20 ± 6.02	<0.001
Pantothenic acid (mg)	5.92 ± 1.92	5.72 ± 1.92	0.264	4.18 ± 1.12	5.36 ± 1.09	6.26 ± 1.23	7.86 ± 1.87	<0.001	5.06 ± 1.80	5.45 ± 1.60	5.98 ± 1.69	7.16 ± 1.88	<0.001
Vitamin B6 (µg)	9.16 ± 5.43	9.20 ± 5.15	0.949	7.34 ± 3.99	8.56 ± 4.63	9.39 ± 5.15	11.36 ± 6.73	<0.001	9.17 ± 5.72	9.18 ± 5.48	8.91 ± 5.19	9.41 ± 5.28	0.5
Folate (µg)	379.45 ± 134.40	369.25 ± 149.57	0.430	282.37 ± 84.74	343.31 ± 87.14	395.93 ± 99.31	495.20 ± 154.05	<0.001	334.94 ± 141.94	345.48 ± 111.83	381.29 ± 119.74	455.05 ± 130.09	<0.001
Vitamin B12 (µg)	5.99 ± 5.58	5.47 ± 3.16	0.329	3.65 ± 1.73	4.86 ± 1.92	5.88 ± 2.28	9.51 ± 9.55	<0.001	5.91 ± 6.28	5.93 ± 6.13	5.70 ± 3.94	6.36 ± 5.43	0.12
**Amino acids**													
Tryptophan (g)	0.80 ± 0.27	0.77 ± 0.29	0.216	0.60 ± 0.21	0.73 ± 0.20	0.83 ± 0.19	1.04 ± 0.26	<0.001	0.59 ± 0.20	0.72 ± 0.18	0.82 ± 0.18	1.07 ± 0.24	<0.001
Threonine (g)	2.53 ± 0.85	2.43 ± 0.90	0.239	1.79 ± 0.54	2.26 ± 0.52	2.64 ± 0.51	3.43 ± 0.78	<0.001	1.99 ± 0.73	2.31 ± 0.67	2.57 ± 0.67	3.24 ± 0.78	<0.001
Isoleucine (g)	3.08 ± 1.02	2.95 ± 1.12	0.215	2.23 ± 0.71	2.76 ± 0.69	3.20 ± 0.67	4.10 ± 0.95	<0.001	2.35 ± 0.84	2.78 ± 0.76	3.14 ± 0.76	4.01 ± 0.93	<0.001
Leucine (g)	5.28 ± 1.77	5.05 ± 1.90	0.171	3.87 ± 1.28	4.76 ± 1.24	5.49 ± 1.21	6.98 ± 1.66	<0.001	3.96 ± 1.39	4.76 ± 1.26	5.41 ± 1.28	6.97 ± 1.59	<0.001
Lysine (g)	3.78 ± 1.35	3.65 ± 1.47	0.306	2.41 ± 0.62	3.28 ± 0.55	3.97 ± 0.56	5.44 ± 1.19	<0.001	3.27 ± 1.32	3.53 ± 1.22	3.79 ± 1.22	4.51 ± 1.32	<0.001
Methionine (g)	1.46 ± 0.49	1.39 ± 0.53	0.179	1.04 ± 0.33	1.30 ± 0.32	1.52 ± 0.31	1.96 ± 0.46	<0.001	1.13 ± 0.42	1.32 ± 0.37	1.49 ± 0.38	1.89 ± 0.45	<0.001
Cysteine (g)	1.16 ± 0.41	1.11 ± 0.44	0.183	0.94 ± 0.37	1.08 ± 0.36	1.20 ± 0.35	1.42 ± 0.39	<0.001	0.78 ± 0.24	1.02 ± 0.21	1.21 ± 0.21	1.64 ± 0.34	<0.001
Phenylalanine (g)	3.29 ± 1.11	3.14 ± 1.18	0.158	2.50 ± 0.88	3.00 ± 0.85	3.41 ± 0.83	4.24 ± 1.05	<0.001	2.38 ± 0.79	2.94 ± 0.71	3.39 ± 0.72	4.45 ± 0.97	<0.001
Tyrosine (g)	2.29 ± 0.77	2.19 ± 0.82	0.165	1.66 ± 0.53	2.06 ± 0.52	2.39 ± 0.51	3.05 ± 0.71	<0.001	1.75 ± 0.62	2.07 ± 0.56	2.35 ± 0.58	2.99 ± 0.70	<0.001
Valine (g)	3.62 ± 1.19	3.46 ± 1.27	0.162	2.62 ± 0.82	3.27 ± 0.80	3.78 ± 0.78	4.82 ± 1.11	<0.001	2.78 ± 0.97	3.28 ± 0.88	3.71 ± 0.90	4.71 ± 1.09	<0.001
Arginine (g)	3.62 ± 1.23	3.51 ± 1.34	0.350	2.51 ± 0.72	3.20 ± 0.70	3.77 ± 0.69	4.98 ± 1.16	<0.001	2.93 ± 1.09	3.31 ± 1.02	3.67 ± 1.03	4.56 ± 1.17	<0.001
Histidine (g)	1.75 ± 0.59	1.68 ± 0.64	0.233	1.25 ± 0.40	1.56 ± 0.38	1.82 ± 0.37	2.35 ± 0.54	<0.001	1.35 ± 0.50	1.58 ± 0.45	1.78 ± 0.45	2.26 ± 0.53	<0.001
Alanine (g)	3.09 ± 1.03	2.97 ± 1.11	0.232	2.17 ± 0.64	2.75 ± 0.61	3.23 ± 0.60	4.22 ± 0.95	<0.001	2.47 ± 0.92	2.83 ± 0.83	3.13 ± 0.83	3.92 ± 0.96	<0.001
Aspartic acid (g)	5.89 ± 1.94	5.69 ± 2.05	0.294	4.00 ± 1.02	5.20 ± 0.94	6.18 ± 0.97	8.14 ± 1.76	<0.001	5.00 ± 1.82	5.47 ± 1.69	5.93 ± 1.72	7.12 ± 1.88	<0.001
Glutamic acid (g)	17.08 ± 6.03	16.24 ± 6.45	0.146	14.08 ± 5.68	15.97 ± 5.40	17.61 ± 5.28	20.57 ± 5.82	<0.001	11.12 ± 3.38	14.90 ± 2.75	17.80 ± 2.85	24.41 ± 4.93	<0.001
Glycine(g)	2.66 ± 0.91	2.56 ± 0.99	0.261	1.93 ± 0.63	2.38 ± 0.61	2.76 ± 0.60	3.57 ± 0.87	<0.001	2.04 ± 0.77	2.41 ± 0.69	2.71 ± 0.68	3.48 ± 0.82	<0.001
Proline (g)	5.56 ± 2.02	5.25 ± 2.21	0.111	4.56 ± 1.89	5.21 ± 1.83	5.74 ± 1.78	6.71 ± 1.97	<0.001	3.55 ± 1.12	4.82 ± 0.91	5.82 ± 0.96	8.02 ± 1.65	<0.001
Serine (g)	3.46 ± 1.15	3.29 ± 1.23	0.134	2.60 ± 0.88	3.15 ± 0.86	3.59 ± 0.84	4.47 ± 1.09	<0.001	2.53 ± 0.83	3.09 ± 0.76	3.56 ± 0.78	4.63 ± 1.02	<0.001

^1^ Data are expressed as means ± SD.

^2^ Student's *t*‐test was used.

^3^ All values were statistically adjusted for sex, age and energy, with the exception of energy intake, which was adjusted solely for sex and age through the application of analysis of covariance.

Table 4 illustrates the associations between dietary AA patterns and the odds of epilepsy. For AAAP, no significant association was observed in the fully adjusted model (OR (Q4 vs. Q1): 0.62, 95% CI: 0.35–1.08, *P* = 0.094), although the *P*‐value for trend was statistically significant (*P*_trend_ = 0.002). In contrast, given to the fourth quartiles compared to the first quartile in the crude model, adherence to the PAAP is associated with a 54% reduction in epilepsy odds (OR (Q4 vs. Q1): 0.46, 95% CI: 0.26–0.81, *P *= 0.008). Moreover, after adjusting the second model based on age and sex, adherence to the PAAP is associated with a 62% reduction in epilepsy odds (OR (Q4 vs. Q1): 0.38, 95% CI: 0.21–0.70, *P *= 0.002). Additionally, adherence to the PAAP is associated with a 63% reduction in epilepsy odds after adjusting based on age, sex, education level, BMI, diabetes, hypertension, marital status, job and any cancer (OR (Q4 vs. Q1): 0.37, 95% CI: 0.20–0.67, *P *= 0.001). In model 3, a cubic spline was fitted to characterize the relationship between AA patterns and the odds of epilepsy (Figure 3).

**TABLE 4:** Odds ratios (ORs) and 95% confidence intervals (95% CIs) for the odds of epilepsy stratified by quartiles of dietary amino acid patterns.

	Quartiles							
Dietary amino acid pattern	Q1 OR (95% CI)	Q2 OR (95% CI)	*P*	Q3 OR (95% CI)	*P*	Q4 OR (95% CI)	*P*	*P*‐trend
**Animal amino‐acid pattern**								
Model 1	Reference	0.82 (0.49, 1.36)	0.440	0.76 (0.45, 1.27)	0.296	0.70 (0.41, 1.18)	0.186	0.572
Model 2	Reference	0.79 (0.47, 1.32)	0.378	0.72 (0.42, 1.22)	0.223	0.64 (0.36, 1.10)	0.108	0.612
Model 3	Reference	0.76 (0.45, 1.28)	0.303	0.74 (0.43, 1.26)	0.263	0.62 (0.35, 1.08)	0.094	**0.002**
**Plant amino‐acid pattern**								
Model 1	Reference	0.60 (0.35, 1.00)	0.054	0.86 (0.54, 1.39)	0.546	0.46 (0.26, 0.81)	**0.008**	**0.025**
Model 2	Reference	0.58 (0.34, 0.97)	0.041	0.79 (0.48, 1.28)	0.338	0.38 (0.21, 0.70)	**0.002**	**0.028**
Model 3	Reference	0.59 (0.34, 0.99)	**0.050**	0.81 (0.49, 1.32)	0.391	0.37 (0.20, 0.67)	**0.001**	**0.001**

Binary logistic regression analysis was employed to derive ORs and their corresponding 95% CIs. The overarching trend of the OR across ascending quantiles was investigated by regarding the median score within each quantile as a continuous predictor variable. *P*‐values shown in bold indicate statistical significance. Model 1: crude model; Model 2: adjusted for sex and age; Model 3: additional adjustments were applied for education level, body mass index, diabetes, hypertension, marital status, job and any cancer.

**FIGURE 3:**
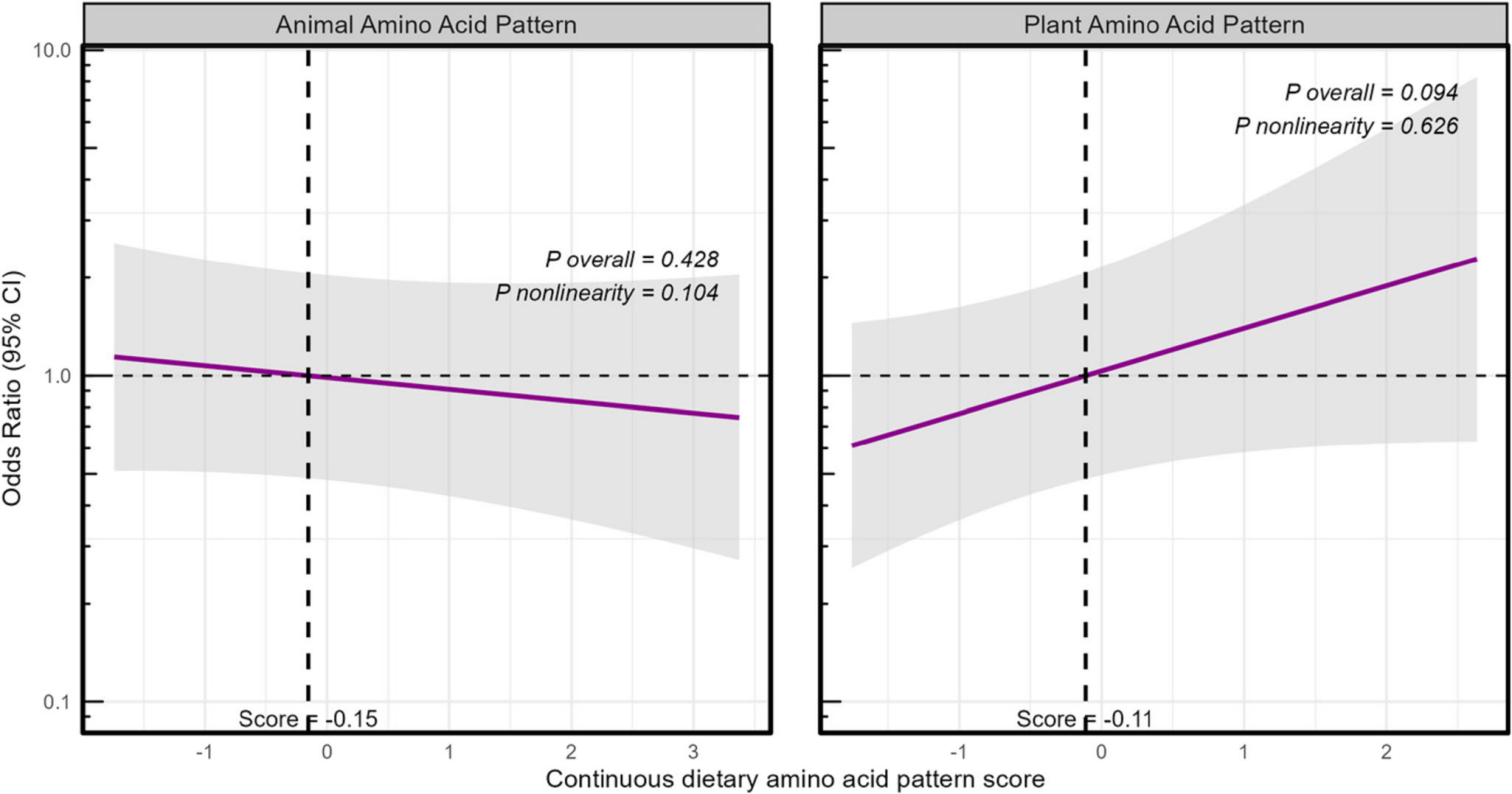
Restricted cubic spline curve of amino acid patterns and the odds of having epilepsy in the fully adjusted model. The *y*‐axis shows odds ratios (ORs) with the shaded area representing 95% CIs.

## 4 DISCUSSION

Our cross‐sectional study reveals a significant inverse association between PAAP and the odds of having epilepsy, with adherence to the highest quartile of this pattern associated with a 63% lower odds of having epilepsy after adjusting for potential confounders, while AAAP only showed a significant inverse trend across quartiles, suggesting a potential linear dose–response association. These findings represent the first comprehensive investigation into dietary AA patterns and the odds of having epilepsy in a cross‐sectional study from the PERSIAN cohort.

The inverse association between PAAP and the odds of having epilepsy could be related to several biological mechanisms, consistent with previous research on specific AAs and seizure control (Gruenbaum et al., 2019; Plaitakis, 1989; Plaitakis et al., 1988). First, the specific combination of AAs within this pattern likely enhances GABAergic neurotransmission while reducing glutamatergic excitation. This mechanism reduces neuronal excitability and subsequent seizure susceptibility. For instance, Gruenbaum's systematic review showed that BCAAs exert anticonvulsant effects by modulating glutamate metabolism and enhancing GABAergic neurotransmission (Gruenbaum et al., 2019). Similarly, studies have highlighted that BCAAs facilitate the clearance of glutamate and favour GABA production by inhibiting glutamate dehydrogenase activity (Plaitakis, 1989; Plaitakis et al., 1988). Jirapinyo et al. (2004) found that patients following a ketogenic diet showed an increased plasma BCAA:aromatic AA (ARAA) ratio, coinciding with a reduced seizure frequency. Second, PAAP may influence glucose homeostasis, as BCAAs are involved in critical nutrient signalling pathways in the hypothalamus and play an important role in maintaining metabolic balance (Biswas et al., 2019). Both hyper‐ and hypoglycaemia can trigger seizure activity, suggesting that AA‐mediated glucose regulation may contribute to seizure control (Urushidani et al., 2024; Huang et al., 2008). Our study extends these findings by identifying comprehensive AA patterns rather than isolated AAs, providing a more holistic understanding of dietary influences on the odds of having epilepsy. The differential associations observed between both AA patterns suggest that the specific composition and relative proportions of AAs within dietary patterns may be relevant for their neuroprotective potential.

Furthermore, the ratio of BCAAs to ARAAs influences central neurotransmitter levels by affecting AA transport across the blood–brain barrier (Sperringer et al., 2017). Additionally, our findings may relate to Sariego‐Jamardo's observation of differential AA profiles in ketogenic diet responders (Sariego‐Jamardo et al., 2015), potentially influencing nitric oxide synthesis and neuromodulation. The comprehensive reduction in both macronutrient and micronutrient intake among epilepsy patients, including vitamins essential for AA metabolism (such as B vitamins), further underscores the importance of overall dietary quality in epilepsy management. These mechanisms collectively suggest that PAAP exerts its protective effect through multiple pathways affecting neurotransmitter balance, cerebral metabolism and neuronal excitability.

The observed trend for AAAP, while not reaching statistical significance for individual quartiles, suggests a potential gradual inverse association that warrants further investigation. The distinction between the two patterns highlights the complexity of dietary AA composition and its relationship with the odds of having epilepsy. Future research should focus on characterizing the specific AA profiles that differentiate these patterns and investigating whether certain AA combinations are more effective than others in conferring neuroprotection.

### 4.1 Strengths and limitations

This study has several methodological and analytical strengths that enhance its scientific rigor and translational relevance. The application of PCA to derive dietary AA patterns represents an innovative approach, capturing the synergistic effects of AAs rather than isolating individual AAs, which aligns with the complex interplay of dietary components in real‐world settings. Furthermore, the use of validated tools, such as the semi‐quantitative FFQ tailored to Iranian dietary habits, ensures reliable and culturally relevant data collection.

Despite these strengths, several limitations warrant consideration. The cross‐sectional nature of our study precludes causal inference, as we cannot establish the temporal sequence between dietary patterns and epilepsy onset. Potential recall bias exists, as the FFQ relies on self‐reported intake over the preceding year, which may not fully capture temporal variations or accurately reflect long‐term dietary habits. Although we adjusted for key confounders, unmeasured variables such as genetic predisposition, medication use, epilepsy severity, seizure frequency, environmental exposures or undiagnosed comorbidities could influence the observed associations. The study's focus on a specific age group (35–70 years) and geographic region (Sabzevar) limits generalizability to younger populations, paediatric epilepsy patients or other ethnic cohorts with different dietary patterns. Some confounders, including age of diagnosis, epilepsy duration, seizure frequency and epilepsy subtype, were not gathered in our cohort data, which make our findings ungeneralizable to all epilepsy subtypes or populations with different dietary habits or genetic backgrounds, as Sariego‐Jamardo et al. (2015) found that responses to dietary interventions may vary based on underlying metabolic factors. The exclusion of individuals with kidney disease and those taking AA supplements may restrict the applicability of findings to these populations.

### 4.2 Implications and future directions

Our findings have relevant clinical and public health implications. The observation of 63% lower odds of having epilepsy in the highest quartile of PAAP supports the exploration of dietary interventions targeting specific AA ratios as complementary approaches to epilepsy management, pending confirmation in prospective studies and randomized controlled trials. Healthcare providers might consider recommending diets rich in beneficial AA profiles, particularly for patients with refractory epilepsy or those experiencing side effects from antiepileptic medications. The observation that individuals with epilepsy consume significantly lower amounts of all assessed AAs highlights the importance of adequate dietary protein intake and AA diversity in this population. Public health recommendations could incorporate guidance on AA‐balanced diets as part of broader epilepsy prevention strategies, particularly in populations with high prevalence of malnutrition or inadequate protein intake, though causality remains to be established.

Future research should prioritize prospective cohort studies and randomized controlled trials to establish causality between dietary AA patterns and the odds of having epilepsy. Longitudinal studies tracking dietary patterns over time and their relationship with epilepsy incidence would provide stronger evidence for temporal relationships. Mechanistic studies exploring the specific roles of individual AAs within protective patterns, their interactions and their effects on neurotransmitter metabolism, neuronal excitability and seizure thresholds are needed. Gruenbaum et al. (2019) emphasized that optimal dosing and duration of BCAA treatment, along with identification of epilepsy conditions that might benefit the most, remain unknown.

Investigation into the biochemical and metabolic differences between both AA patterns could elucidate why one pattern demonstrates stronger protective associations than the other. Studies examining whether dietary interventions based on these patterns can reduce seizure frequency or severity in individuals with existing epilepsy would have immediate clinical relevance. Given the lower micronutrient intake observed in individuals with epilepsy, studies examining the combined effects of AA patterns and micronutrient status on epilepsy odds would provide valuable insights.

Personalized approaches based on individual metabolic profiles, genetic factors (such as polymorphisms affecting AA metabolism) and clinical characteristics (epilepsy type, seizure frequency, medication regimen) should be explored. Investigation into how sociodemographic factors, lifestyle variables and comorbidities modify the relationship between dietary AA patterns and epilepsy could inform targeted interventions for specific subgroups. Finally, cross‐cultural studies examining whether similar AA patterns are associated with epilepsy odds in populations with different dietary habits would enhance the generalizability of our findings.

### 4.3 Conclusion

This study suggests that dietary AA patterns are associated with the odds of having epilepsy. The inverse association observed between adherence to PAAP and the odds of having epilepsy underscores a possible role of specific AA combinations, particularly those rich in proline, glutamic acid, cysteine, phenylalanine, serine and tryptophan, while AAAP did not show a significant overall association. These findings not only advance our understanding of dietary influences on neurological health but also emphasize the need for including the key role of AAs in preventive dietary guidelines for epilepsy prevention.

## AUTHOR CONTRIBUTIONS

Hamid Abbasi: drafting the original manuscript, preparing figures, data curation, formal analysis; Ali Jafari: drafting the original manuscript; Saeid Doaei, Akram Kooshki and Raul Zamora‐Ros: conceptualization, supervision, and critically editing the manuscript. All authors have read and approved the final version of this manuscript and agree to be accountable for all aspects of the work in ensuring that questions related to the accuracy or integrity of any part of the work are appropriately investigated and resolved. All persons designated as authors qualify for authorship, and all those who qualify for authorship are listed.

## CONFLICT OF INTEREST

None declared.

## GENERATIVE AI STATEMENT

The authors utilized OpenAI ChatGPT (GPT 5.5) for language editing and enhancing the text while revising the manuscript. All scientific content, interpretations and conclusions have been thoroughly reviewed and approved by the authors, who assume complete responsibility for the final version of the manuscript.

## Supporting information



1. Tables S1‐S3.

2. STROBE Statement.

## Data Availability

The data presented in this study are available on request from the corresponding author.
