# Supplementary material for: Dietary amino acid patterns and epilepsy among the PERSIAN cohort population: A cross‐sectional study
Source: Exp Physiol. 2026 Oct 2:10.1113/EP093525. Online ahead of print. doi: 10.1113/EP093525 (PMC13632661; doi:10.1113/EP093525)
Supplement: Supplementary file 1 [file EPH-9999-0-s001.docx]

Table S1. Significant pairwise differences for continuous variables (Tukey HSD).

| **Pattern** | **Variable** | **Comparison** | **Difference** | **95% CI lower** | **95% CI upper** | **Adjusted p** |
| --- | --- | --- | --- | --- | --- | --- |
| AAAP | Age | 2-1 | -1.04 | -2.03 | -0.05 | 0.03427 |
| AAAP | Age | 3-1 | -2.12 | -3.11 | -1.13 | < 0.001 |
| AAAP | Age | 4-1 | -3.44 | -4.43 | -2.45 | < 0.001 |
| AAAP | Age | 3-2 | -1.08 | -2.07 | -0.09 | 0.02556 |
| AAAP | Age | 4-2 | -2.40 | -3.39 | -1.41 | < 0.001 |
| AAAP | Age | 4-3 | -1.32 | -2.31 | -0.33 | 0.00337 |
| PAAP | Age | 4-1 | -1.39 | -2.39 | -0.39 | 0.00196 |
| PAAP | Age | 4-2 | -1.15 | -2.15 | -0.16 | 0.01564 |
| PAAP | Age | 4-3 | -1.58 | -2.58 | -0.59 | < 0.001 |

Table S2. Significant pairwise differences for categorical variables (Bonferroni-adjusted proportion tests).

| **Pattern** | **Variable** | **Category** | **Comparison** | **Adjusted p** |
| --- | --- | --- | --- | --- |
| AAAP | Gender | Male | 1 vs 2 | < 0.001 |
| AAAP | Gender | Male | 1 vs 3 | < 0.001 |
| AAAP | Gender | Male | 1 vs 4 | < 0.001 |
| AAAP | Gender | Male | 2 vs 4 | < 0.001 |
| AAAP | Gender | Male | 3 vs 4 | < 0.001 |
| AAAP | Gender | Female | 1 vs 2 | < 0.001 |
| AAAP | Gender | Female | 1 vs 3 | < 0.001 |
| AAAP | Gender | Female | 1 vs 4 | < 0.001 |
| AAAP | Gender | Female | 2 vs 4 | < 0.001 |
| AAAP | Gender | Female | 3 vs 4 | < 0.001 |
| AAAP | Education | Elementary School | 1 vs 3 | < 0.001 |
| AAAP | Education | Elementary School | 1 vs 4 | < 0.001 |
| AAAP | Education | Elementary School | 2 vs 4 | < 0.001 |
| AAAP | Education | Elementary School | 3 vs 4 | 0.03492 |
| AAAP | Education | High School diploma | 1 vs 3 | 0.00964 |
| AAAP | Education | High School diploma | 1 vs 4 | < 0.001 |
| AAAP | Education | Associate degree | 1 vs 3 | < 0.001 |
| AAAP | Education | Associate degree | 1 vs 4 | 0.01662 |
| AAAP | Education | Bachelor’s degree | 1 vs 2 | < 0.001 |
| AAAP | Education | Bachelor’s degree | 1 vs 3 | < 0.001 |
| AAAP | Education | Bachelor’s degree | 1 vs 4 | < 0.001 |
| AAAP | Education | Bachelor’s degree | 2 vs 4 | 0.01042 |
| AAAP | Education | Master’s degree | 1 vs 3 | 0.01632 |
| AAAP | Education | Master’s degree | 1 vs 4 | < 0.001 |
| AAAP | Education | Illiterate | 1 vs 2 | < 0.001 |
| AAAP | Education | Illiterate | 1 vs 3 | < 0.001 |
| AAAP | Education | Illiterate | 1 vs 4 | < 0.001 |
| AAAP | Education | Illiterate | 2 vs 4 | < 0.001 |
| AAAP | Marital Status | Married | 1 vs 2 | 0.02930 |
| AAAP | Marital Status | Married | 1 vs 3 | < 0.001 |
| AAAP | Marital Status | Married | 1 vs 4 | < 0.001 |
| AAAP | Marital Status | Widowed | 1 vs 2 | 0.00870 |
| AAAP | Marital Status | Widowed | 1 vs 3 | < 0.001 |
| AAAP | Marital Status | Widowed | 1 vs 4 | < 0.001 |
| AAAP | Job | No | 1 vs 3 | < 0.001 |
| AAAP | Job | No | 1 vs 4 | < 0.001 |
| AAAP | Job | No | 2 vs 3 | 0.00850 |
| AAAP | Job | No | 2 vs 4 | < 0.001 |
| AAAP | Job | No | 3 vs 4 | < 0.001 |
| AAAP | Job | Yes | 1 vs 3 | < 0.001 |
| AAAP | Job | Yes | 1 vs 4 | < 0.001 |
| AAAP | Job | Yes | 2 vs 3 | 0.00850 |
| AAAP | Job | Yes | 2 vs 4 | < 0.001 |
| AAAP | Job | Yes | 3 vs 4 | < 0.001 |
| AAAP | Hypertension | No | 1 vs 4 | < 0.001 |
| AAAP | Hypertension | Yes | 1 vs 4 | < 0.001 |
| AAAP | Smoking | Yes | 1 vs 4 | < 0.001 |
| AAAP | Smoking | Yes | 2 vs 4 | < 0.001 |
| AAAP | Smoking | Yes | 3 vs 4 | 0.01964 |
| AAAP | Smoking | No | 1 vs 4 | < 0.001 |
| AAAP | Smoking | No | 2 vs 4 | < 0.001 |
| AAAP | Smoking | No | 3 vs 4 | 0.02016 |
| AAAP | Alcohol | No | 1 vs 4 | < 0.001 |
| AAAP | Alcohol | No | 2 vs 4 | < 0.001 |
| AAAP | Alcohol | No | 3 vs 4 | < 0.001 |
| AAAP | Alcohol | Yes | 1 vs 4 | < 0.001 |
| AAAP | Alcohol | Yes | 2 vs 4 | < 0.001 |
| AAAP | Alcohol | Yes | 3 vs 4 | < 0.001 |
| PAAP | Gender | Male | 1 vs 2 | 0.00160 |
| PAAP | Gender | Male | 1 vs 3 | < 0.001 |
| PAAP | Gender | Male | 1 vs 4 | < 0.001 |
| PAAP | Gender | Male | 2 vs 3 | < 0.001 |
| PAAP | Gender | Male | 2 vs 4 | < 0.001 |
| PAAP | Gender | Male | 3 vs 4 | < 0.001 |
| PAAP | Gender | Female | 1 vs 2 | 0.00160 |
| PAAP | Gender | Female | 1 vs 3 | < 0.001 |
| PAAP | Gender | Female | 1 vs 4 | < 0.001 |
| PAAP | Gender | Female | 2 vs 3 | < 0.001 |
| PAAP | Gender | Female | 2 vs 4 | < 0.001 |
| PAAP | Gender | Female | 3 vs 4 | < 0.001 |
| PAAP | Marital Status | Married | 1 vs 4 | < 0.001 |
| PAAP | Marital Status | Married | 2 vs 4 | 0.01093 |
| PAAP | Marital Status | Widowed | 1 vs 4 | < 0.001 |
| PAAP | Job | No | 1 vs 3 | < 0.001 |
| PAAP | Job | No | 1 vs 4 | < 0.001 |
| PAAP | Job | No | 2 vs 3 | < 0.001 |
| PAAP | Job | No | 2 vs 4 | < 0.001 |
| PAAP | Job | No | 3 vs 4 | < 0.001 |
| PAAP | Job | Yes | 1 vs 3 | < 0.001 |
| PAAP | Job | Yes | 1 vs 4 | < 0.001 |
| PAAP | Job | Yes | 2 vs 3 | < 0.001 |
| PAAP | Job | Yes | 2 vs 4 | < 0.001 |
| PAAP | Job | Yes | 3 vs 4 | < 0.001 |
| PAAP | Hypertension | No | 1 vs 4 | 0.01652 |
| PAAP | Hypertension | Yes | 1 vs 4 | 0.01652 |
| PAAP | Smoking | Yes | 1 vs 3 | 0.01179 |
| PAAP | Smoking | Yes | 1 vs 4 | < 0.001 |
| PAAP | Smoking | Yes | 2 vs 4 | < 0.001 |
| PAAP | Smoking | No | 1 vs 3 | 0.00941 |
| PAAP | Smoking | No | 1 vs 4 | < 0.001 |
| PAAP | Smoking | No | 2 vs 4 | < 0.001 |
| PAAP | Alcohol | No | 1 vs 4 | 0.00339 |
| PAAP | Alcohol | No | 3 vs 4 | 0.01004 |
| PAAP | Alcohol | Yes | 1 vs 4 | 0.00339 |
| PAAP | Alcohol | Yes | 3 vs 4 | 0.01004 |

***Table S3. Significant pairwise differences for nutrients (Tukey HSD).***

| **Pattern** | **Variable** | **Comparison** | **Estimate** | **Lower.CL** | **Upper.CL** | **P value** |
| --- | --- | --- | --- | --- | --- | --- |
| AAAP | Alanine | 1-2 | -0.27211539 | -0.319835763 | -0.224395025 | <0.001 |
| AAAP | Alanine | 1-3 | -0.50297659 | -0.552527509 | -0.453425680 | <0.001 |
| AAAP | Alanine | 1-4 | -1.06286749 | -1.117626056 | -1.008108920 | <0.001 |
| AAAP | Alanine | 2-3 | -0.23086120 | -0.278325978 | -0.183396425 | <0.001 |
| AAAP | Alanine | 2-4 | -0.79075209 | -0.841551573 | -0.739952616 | <0.001 |
| AAAP | Alanine | 3-4 | -0.55989089 | -0.608404303 | -0.511377483 | <0.001 |
| AAAP | Arginine | 1-2 | -0.32327587 | -0.379057310 | -0.267494426 | <0.001 |
| AAAP | Arginine | 1-3 | -0.59998085 | -0.657902064 | -0.542059646 | <0.001 |
| AAAP | Arginine | 1-4 | -1.30035168 | -1.364360232 | -1.236343120 | <0.001 |
| AAAP | Arginine | 2-3 | -0.27670499 | -0.332187661 | -0.221222313 | <0.001 |
| AAAP | Arginine | 2-4 | -0.97707581 | -1.036456492 | -0.917695123 | <0.001 |
| AAAP | Arginine | 3-4 | -0.70037082 | -0.757079267 | -0.643662375 | <0.001 |
| AAAP | Aspartic_Acid | 1-2 | -0.69071023 | -0.774426680 | -0.606993787 | <0.001 |
| AAAP | Aspartic_Acid | 1-3 | -1.25247468 | -1.339402478 | -1.165546887 | <0.001 |
| AAAP | Aspartic_Acid | 1-4 | -2.49029724 | -2.586360889 | -2.394233592 | <0.001 |
| AAAP | Aspartic_Acid | 2-3 | -0.56176445 | -0.645032506 | -0.478496391 | <0.001 |
| AAAP | Aspartic_Acid | 2-4 | -1.79958701 | -1.888705174 | -1.710468839 | <0.001 |
| AAAP | Aspartic_Acid | 3-4 | -1.23782256 | -1.322930246 | -1.152714870 | <0.001 |
| AAAP | Carbohydrate | 1-2 | 15.36742962 | 11.498341605 | 19.236517633 | <0.001 |
| AAAP | Carbohydrate | 1-3 | 26.20349779 | 22.185992200 | 30.221003381 | <0.001 |
| AAAP | Carbohydrate | 1-4 | 56.72821548 | 52.288482020 | 61.167948950 | <0.001 |
| AAAP | Carbohydrate | 2-3 | 10.83606817 | 6.987703182 | 14.684433161 | <0.001 |
| AAAP | Carbohydrate | 2-4 | 41.36078587 | 37.242048771 | 45.479522962 | <0.001 |
| AAAP | Carbohydrate | 3-4 | 30.52471769 | 26.591331264 | 34.458104125 | <0.001 |
| AAAP | Cysteine | 1-2 | 0.02413681 | 0.001335994 | 0.046937634 | 0.033 |
| AAAP | Cysteine | 1-3 | 0.03615816 | 0.012482705 | 0.059833617 | <0.001 |
| AAAP | Cysteine | 1-4 | 0.04111329 | 0.014949614 | 0.067276965 | <0.001 |
| AAAP | Energy | 1-2 | -290.50190690 | -361.502667366 | -219.501146438 | <0.001 |
| AAAP | Energy | 1-3 | -538.88791610 | -610.376437171 | -467.399395034 | <0.001 |
| AAAP | Energy | 1-4 | -936.36935610 | -1,009.793850143 | -862.944862055 | <0.001 |
| AAAP | Energy | 2-3 | -248.38600920 | -319.257271982 | -177.514746419 | <0.001 |
| AAAP | Energy | 2-4 | -645.86744920 | -717.903359936 | -573.831538458 | <0.001 |
| AAAP | Energy | 3-4 | -397.48144000 | -468.859477768 | -326.103402225 | <0.001 |
| AAAP | Folate_total | 1-2 | -19.79067645 | -28.615683103 | -10.965669806 | <0.001 |
| AAAP | Folate_total | 1-3 | -38.50953598 | -47.673068421 | -29.346003533 | <0.001 |
| AAAP | Folate_total | 1-4 | -80.30781796 | -90.434410377 | -70.181225541 | <0.001 |
| AAAP | Folate_total | 2-3 | -18.71885952 | -27.496599003 | -9.941120041 | <0.001 |
| AAAP | Folate_total | 2-4 | -60.51714150 | -69.911572988 | -51.122710021 | <0.001 |
| AAAP | Folate_total | 3-4 | -41.79828198 | -50.769946952 | -32.826617012 | <0.001 |
| AAAP | Glutamic_Acid | 1-2 | 0.55729293 | 0.214435019 | 0.900150834 | <0.001 |
| AAAP | Glutamic_Acid | 1-3 | 0.89214903 | 0.536139149 | 1.248158909 | <0.001 |
| AAAP | Glutamic_Acid | 1-4 | 1.38890814 | 0.995482685 | 1.782333602 | <0.001 |
| AAAP | Glutamic_Acid | 2-4 | 0.83161522 | 0.466634742 | 1.196595692 | <0.001 |
| AAAP | Glutamic_Acid | 3-4 | 0.49675911 | 0.148203425 | 0.845314804 | 0.001 |
| AAAP | Glycine | 1-2 | -0.15633701 | -0.202276518 | -0.110397502 | <0.001 |
| AAAP | Glycine | 1-3 | -0.30202500 | -0.349726735 | -0.254323256 | <0.001 |
| AAAP | Glycine | 1-4 | -0.69326994 | -0.745984987 | -0.640554886 | <0.001 |
| AAAP | Glycine | 2-3 | -0.14568799 | -0.191381439 | -0.099994532 | <0.001 |
| AAAP | Glycine | 2-4 | -0.53693293 | -0.585836636 | -0.488029217 | <0.001 |
| AAAP | Glycine | 3-4 | -0.39124494 | -0.437947894 | -0.344541988 | <0.001 |
| AAAP | Histidine | 1-2 | -0.12933764 | -0.158108973 | -0.100566304 | <0.001 |
| AAAP | Histidine | 1-3 | -0.23879603 | -0.268671032 | -0.208921037 | <0.001 |
| AAAP | Histidine | 1-4 | -0.51354055 | -0.546555324 | -0.480525782 | <0.001 |
| AAAP | Histidine | 2-3 | -0.10945840 | -0.138075629 | -0.080841162 | <0.001 |
| AAAP | Histidine | 2-4 | -0.38420291 | -0.414830691 | -0.353575139 | <0.001 |
| AAAP | Histidine | 3-4 | -0.27474452 | -0.303993989 | -0.245495048 | <0.001 |
| AAAP | Isoleucine | 1-2 | -0.21012565 | -0.260888586 | -0.159362724 | <0.001 |
| AAAP | Isoleucine | 1-3 | -0.38078341 | -0.433493597 | -0.328073220 | <0.001 |
| AAAP | Isoleucine | 1-4 | -0.82069862 | -0.878948493 | -0.762448748 | <0.001 |
| AAAP | Isoleucine | 2-3 | -0.17065775 | -0.221148796 | -0.120166711 | <0.001 |
| AAAP | Isoleucine | 2-4 | -0.61057297 | -0.664611325 | -0.556534607 | <0.001 |
| AAAP | Isoleucine | 3-4 | -0.43991521 | -0.491521747 | -0.388308678 | <0.001 |
| AAAP | Leucine | 1-2 | -0.30546394 | -0.393726194 | -0.217201678 | <0.001 |
| AAAP | Leucine | 1-3 | -0.55360212 | -0.645250104 | -0.461954137 | <0.001 |
| AAAP | Leucine | 1-4 | -1.21150080 | -1.312780709 | -1.110220881 | <0.001 |
| AAAP | Leucine | 2-3 | -0.24813818 | -0.335927706 | -0.160348664 | <0.001 |
| AAAP | Leucine | 2-4 | -0.90603686 | -0.999994152 | -0.812079567 | <0.001 |
| AAAP | Leucine | 3-4 | -0.65789867 | -0.747627718 | -0.568169631 | <0.001 |
| AAAP | Lysine | 1-2 | -0.58585401 | -0.651411700 | -0.520296323 | <0.001 |
| AAAP | Lysine | 1-3 | -1.05067448 | -1.118746955 | -0.982602014 | <0.001 |
| AAAP | Lysine | 1-4 | -2.12624248 | -2.201469160 | -2.051015791 | <0.001 |
| AAAP | Lysine | 2-3 | -0.46482047 | -0.530027031 | -0.399613914 | <0.001 |
| AAAP | Lysine | 2-4 | -1.54038846 | -1.610176198 | -1.470600730 | <0.001 |
| AAAP | Lysine | 3-4 | -1.07556799 | -1.142215150 | -1.008920833 | <0.001 |
| AAAP | Methionine | 1-2 | -0.11612419 | -0.141352745 | -0.090895630 | <0.001 |
| AAAP | Methionine | 1-3 | -0.21078161 | -0.236977927 | -0.184585286 | <0.001 |
| AAAP | Methionine | 1-4 | -0.44223128 | -0.471180754 | -0.413281803 | <0.001 |
| AAAP | Methionine | 2-3 | -0.09465742 | -0.119750851 | -0.069563988 | <0.001 |
| AAAP | Methionine | 2-4 | -0.32610709 | -0.352963495 | -0.299250686 | <0.001 |
| AAAP | Methionine | 3-4 | -0.23144967 | -0.257097489 | -0.205801854 | <0.001 |
| AAAP | Niacin | 1-2 | -0.69106175 | -1.027676711 | -0.354446780 | <0.001 |
| AAAP | Niacin | 1-3 | -1.54275519 | -1.892282650 | -1.193227731 | <0.001 |
| AAAP | Niacin | 1-4 | -3.61550243 | -4.001764183 | -3.229240673 | <0.001 |
| AAAP | Niacin | 2-3 | -0.85169345 | -1.186505485 | -0.516881406 | <0.001 |
| AAAP | Niacin | 2-4 | -2.92444068 | -3.282775396 | -2.566105969 | <0.001 |
| AAAP | Niacin | 3-4 | -2.07274724 | -2.414956237 | -1.730538238 | <0.001 |
| AAAP | Pantothenic_Acid | 1-2 | -0.58749052 | -0.675092694 | -0.499888348 | <0.001 |
| AAAP | Pantothenic_Acid | 1-3 | -1.00492701 | -1.095889585 | -0.913964428 | <0.001 |
| AAAP | Pantothenic_Acid | 1-4 | -1.78987047 | -1.890392949 | -1.689348000 | <0.001 |
| AAAP | Pantothenic_Acid | 2-3 | -0.41743649 | -0.504569458 | -0.330303514 | <0.001 |
| AAAP | Pantothenic_Acid | 2-4 | -1.20237995 | -1.295634571 | -1.109125336 | <0.001 |
| AAAP | Pantothenic_Acid | 3-4 | -0.78494347 | -0.874001457 | -0.695885478 | <0.001 |
| AAAP | Phenylalanine | 1-2 | -0.10234060 | -0.158590971 | -0.046090222 | <0.001 |
| AAAP | Phenylalanine | 1-3 | -0.19019329 | -0.248601421 | -0.131785161 | <0.001 |
| AAAP | Phenylalanine | 1-4 | -0.45789527 | -0.522441921 | -0.393348620 | <0.001 |
| AAAP | Phenylalanine | 2-3 | -0.08785269 | -0.143801790 | -0.031903600 | <0.001 |
| AAAP | Phenylalanine | 2-4 | -0.35555467 | -0.415434549 | -0.295674799 | <0.001 |
| AAAP | Phenylalanine | 3-4 | -0.26770198 | -0.324887151 | -0.210516808 | <0.001 |
| AAAP | Proline | 1-2 | 0.16631053 | 0.049337551 | 0.283283509 | 0.001 |
| AAAP | Proline | 1-3 | 0.30287477 | 0.181414724 | 0.424334811 | <0.001 |
| AAAP | Proline | 1-4 | 0.49194830 | 0.357723167 | 0.626173432 | <0.001 |
| AAAP | Proline | 2-3 | 0.13656424 | 0.020217772 | 0.252910704 | 0.014 |
| AAAP | Proline | 2-4 | 0.32563777 | 0.201117225 | 0.450158315 | <0.001 |
| AAAP | Proline | 3-4 | 0.18907353 | 0.070156638 | 0.307990426 | <0.001 |
| AAAP | Riboflavin | 1-2 | -0.16721406 | -0.206963873 | -0.127464252 | <0.001 |
| AAAP | Riboflavin | 1-3 | -0.28742290 | -0.328697502 | -0.246148288 | <0.001 |
| AAAP | Riboflavin | 1-4 | -0.61470168 | -0.660314123 | -0.569089233 | <0.001 |
| AAAP | Riboflavin | 2-3 | -0.12020883 | -0.159745741 | -0.080671924 | <0.001 |
| AAAP | Riboflavin | 2-4 | -0.44748762 | -0.489802243 | -0.405172987 | <0.001 |
| AAAP | Riboflavin | 3-4 | -0.32727878 | -0.367689175 | -0.286868390 | <0.001 |
| AAAP | Serine | 1-2 | -0.14025152 | -0.197033392 | -0.083469642 | <0.001 |
| AAAP | Serine | 1-3 | -0.24797601 | -0.306936029 | -0.189015992 | <0.001 |
| AAAP | Serine | 1-4 | -0.54973818 | -0.614894726 | -0.484581644 | <0.001 |
| AAAP | Serine | 2-3 | -0.10772449 | -0.164202242 | -0.051246745 | <0.001 |
| AAAP | Serine | 2-4 | -0.40948667 | -0.469932337 | -0.349040998 | <0.001 |
| AAAP | Serine | 3-4 | -0.30176217 | -0.359487679 | -0.244036670 | <0.001 |
| AAAP | Thiamin | 1-2 | 0.06994248 | 0.041487093 | 0.098397868 | <0.001 |
| AAAP | Thiamin | 1-3 | 0.11347553 | 0.083928601 | 0.143022464 | <0.001 |
| AAAP | Thiamin | 1-4 | 0.18514360 | 0.152491378 | 0.217795830 | <0.001 |
| AAAP | Thiamin | 2-3 | 0.04353305 | 0.015230072 | 0.071836031 | <0.001 |
| AAAP | Thiamin | 2-4 | 0.11520112 | 0.084909680 | 0.145492566 | <0.001 |
| AAAP | Thiamin | 3-4 | 0.07166807 | 0.042739798 | 0.100596345 | <0.001 |
| AAAP | Threonine | 1-2 | -0.21020069 | -0.250085786 | -0.170315600 | <0.001 |
| AAAP | Threonine | 1-3 | -0.38427524 | -0.425690318 | -0.342860160 | <0.001 |
| AAAP | Threonine | 1-4 | -0.81575611 | -0.861523785 | -0.769988425 | <0.001 |
| AAAP | Threonine | 2-3 | -0.17407455 | -0.213746013 | -0.134403079 | <0.001 |
| AAAP | Threonine | 2-4 | -0.60555541 | -0.648014052 | -0.563096773 | <0.001 |
| AAAP | Threonine | 3-4 | -0.43148087 | -0.472028790 | -0.390932943 | <0.001 |
| AAAP | Total_lipid_fat | 1-2 | -6.50378304 | -8.256589728 | -4.750976362 | <0.001 |
| AAAP | Total_lipid_fat | 1-3 | -10.72823971 | -12.548283771 | -8.908195657 | <0.001 |
| AAAP | Total_lipid_fat | 1-4 | -21.65318294 | -23.664508211 | -19.641857674 | <0.001 |
| AAAP | Total_lipid_fat | 2-3 | -4.22445667 | -5.967875234 | -2.481038105 | <0.001 |
| AAAP | Total_lipid_fat | 2-4 | -15.14939990 | -17.015304700 | -13.283495095 | <0.001 |
| AAAP | Total_lipid_fat | 3-4 | -10.92494323 | -12.706878919 | -9.143007538 | <0.001 |
| AAAP | Tryptophan | 1-2 | -0.02927120 | -0.042843646 | -0.015698759 | <0.001 |
| AAAP | Tryptophan | 1-3 | -0.05601811 | -0.070111195 | -0.041925035 | <0.001 |
| AAAP | Tryptophan | 1-4 | -0.13500964 | -0.150583857 | -0.119435416 | <0.001 |
| AAAP | Tryptophan | 2-3 | -0.02674691 | -0.040246660 | -0.013247163 | <0.001 |
| AAAP | Tryptophan | 2-4 | -0.10573843 | -0.120186626 | -0.091290242 | <0.001 |
| AAAP | Tryptophan | 3-4 | -0.07899152 | -0.092789519 | -0.065193525 | <0.001 |
| AAAP | Tyrosine | 1-2 | -0.16066390 | -0.199135460 | -0.122192341 | <0.001 |
| AAAP | Tyrosine | 1-3 | -0.28652995 | -0.326477272 | -0.246582628 | <0.001 |
| AAAP | Tyrosine | 1-4 | -0.60801655 | -0.652162212 | -0.563870878 | <0.001 |
| AAAP | Tyrosine | 2-3 | -0.12586605 | -0.164131553 | -0.087600545 | <0.001 |
| AAAP | Tyrosine | 2-4 | -0.44735264 | -0.488306543 | -0.406398745 | <0.001 |
| AAAP | Tyrosine | 3-4 | -0.32148660 | -0.360597494 | -0.282375697 | <0.001 |
| AAAP | Valine | 1-2 | -0.25749867 | -0.314772125 | -0.200225223 | <0.001 |
| AAAP | Valine | 1-3 | -0.45649380 | -0.515964247 | -0.397023344 | <0.001 |
| AAAP | Valine | 1-4 | -0.95262784 | -1.018348463 | -0.886907225 | <0.001 |
| AAAP | Valine | 2-3 | -0.19899512 | -0.255961813 | -0.142028429 | <0.001 |
| AAAP | Valine | 2-4 | -0.69512917 | -0.756098134 | -0.634160206 | <0.001 |
| AAAP | Valine | 3-4 | -0.49613405 | -0.554359299 | -0.437908799 | <0.001 |
| AAAP | VitaminA_mcg | 1-2 | -110.93335985 | -155.447945945 | -66.418773753 | <0.001 |
| AAAP | VitaminA_mcg | 1-3 | -200.03409990 | -246.256258155 | -153.811941641 | <0.001 |
| AAAP | VitaminA_mcg | 1-4 | -440.97821779 | -492.058187246 | -389.898248332 | <0.001 |
| AAAP | VitaminA_mcg | 2-3 | -89.10074005 | -133.376903843 | -44.824576254 | <0.001 |
| AAAP | VitaminA_mcg | 2-4 | -330.04485794 | -377.431703725 | -282.658012155 | <0.001 |
| AAAP | VitaminA_mcg | 3-4 | -240.94411789 | -286.198469376 | -195.689766406 | <0.001 |
| AAAP | VitaminB12 | 1-2 | -0.80165181 | -1.383957207 | -0.219346418 | 0.002 |
| AAAP | VitaminB12 | 1-3 | -1.49587148 | -2.100514009 | -0.891228943 | <0.001 |
| AAAP | VitaminB12 | 1-4 | -4.54863337 | -5.216822029 | -3.880444703 | <0.001 |
| AAAP | VitaminB12 | 2-3 | -0.69421966 | -1.273406202 | -0.115033125 | 0.011 |
| AAAP | VitaminB12 | 2-4 | -3.74698155 | -4.366859628 | -3.127103478 | <0.001 |
| AAAP | VitaminB12 | 3-4 | -3.05276189 | -3.644744323 | -2.460779456 | <0.001 |
| AAAP | VitaminB6 | 1-2 | -0.88762519 | -1.486085086 | -0.289165287 | <0.001 |
| AAAP | VitaminB6 | 1-3 | -1.43319794 | -2.054614659 | -0.811781215 | <0.001 |
| AAAP | VitaminB6 | 1-4 | -2.94909305 | -3.635818818 | -2.262367280 | <0.001 |
| AAAP | VitaminB6 | 2-4 | -2.06146786 | -2.698542795 | -1.424392929 | <0.001 |
| AAAP | VitaminB6 | 3-4 | -1.51589511 | -2.124300513 | -0.907489710 | <0.001 |
| AAAP | VitaminC_total_ascorbic_acid | 1-2 | -23.18865096 | -31.082402344 | -15.294899584 | <0.001 |
| AAAP | VitaminC_total_ascorbic_acid | 1-3 | -42.92908558 | -51.125639956 | -34.732531200 | <0.001 |
| AAAP | VitaminC_total_ascorbic_acid | 1-4 | -67.26704236 | -76.325030200 | -58.209054526 | <0.001 |
| AAAP | VitaminC_total_ascorbic_acid | 2-3 | -19.74043461 | -27.591906676 | -11.888962552 | <0.001 |
| AAAP | VitaminC_total_ascorbic_acid | 2-4 | -44.07839140 | -52.481479289 | -35.675303510 | <0.001 |
| AAAP | VitaminC_total_ascorbic_acid | 3-4 | -24.33795679 | -32.362890427 | -16.313023143 | <0.001 |
| AAAP | VitaminD_IU | 1-2 | -7.89758233 | -10.420587416 | -5.374577249 | <0.001 |
| AAAP | VitaminD_IU | 1-3 | -15.21575358 | -17.835540724 | -12.595966443 | <0.001 |
| AAAP | VitaminD_IU | 1-4 | -31.82093859 | -34.716057558 | -28.925819621 | <0.001 |
| AAAP | VitaminD_IU | 2-3 | -7.31817125 | -9.827662995 | -4.808679506 | <0.001 |
| AAAP | VitaminD_IU | 2-4 | -23.92335626 | -26.609155750 | -21.237556763 | <0.001 |
| AAAP | VitaminD_IU | 3-4 | -16.60518501 | -19.170118634 | -14.040251378 | <0.001 |
| AAAP | VitaminE_alpha_tocopheraol | 1-2 | -0.87466026 | -1.148323018 | -0.600997502 | <0.001 |
| AAAP | VitaminE_alpha_tocopheraol | 1-3 | -1.53721662 | -1.821377036 | -1.253056203 | <0.001 |
| AAAP | VitaminE_alpha_tocopheraol | 1-4 | -3.07336518 | -3.387390009 | -2.759340349 | <0.001 |
| AAAP | VitaminE_alpha_tocopheraol | 2-3 | -0.66255636 | -0.934753367 | -0.390359353 | <0.001 |
| AAAP | VitaminE_alpha_tocopheraol | 2-4 | -2.19870492 | -2.490025496 | -1.907384342 | <0.001 |
| AAAP | VitaminE_alpha_tocopheraol | 3-4 | -1.53614856 | -1.814359181 | -1.257937938 | <0.001 |
| AAAP | VitaminK | 1-2 | -29.88297804 | -40.429433487 | -19.336522583 | <0.001 |
| AAAP | VitaminK | 1-3 | -52.12560601 | -63.076621751 | -41.174590273 | <0.001 |
| AAAP | VitaminK | 1-4 | -82.29832029 | -94.400255174 | -70.196385404 | <0.001 |
| AAAP | VitaminK | 2-3 | -22.24262798 | -32.732596097 | -11.752659857 | <0.001 |
| AAAP | VitaminK | 2-4 | -52.41534225 | -63.642297324 | -41.188387184 | <0.001 |
| AAAP | VitaminK | 3-4 | -30.17271428 | -40.894435937 | -19.450992617 | <0.001 |
| AAAP | protein | 1-2 | -3.72497810 | -4.777748916 | -2.672207294 | <0.001 |
| AAAP | protein | 1-3 | -6.93044923 | -8.023604154 | -5.837294304 | <0.001 |
| AAAP | protein | 1-4 | -15.68581094 | -16.893853197 | -14.477768691 | <0.001 |
| AAAP | protein | 2-3 | -3.20547112 | -4.252603244 | -2.158339006 | <0.001 |
| AAAP | protein | 2-4 | -11.96083284 | -13.081532647 | -10.840133032 | <0.001 |
| AAAP | protein | 3-4 | -8.75536171 | -9.825627991 | -7.685095438 | <0.001 |
| PAAP | Alanine | 1-3 | 0.07223517 | 0.007763492 | 0.136706841 | 0.021 |
| PAAP | Alanine | 1-4 | 0.09268291 | 0.016110759 | 0.169255068 | 0.010 |
| PAAP | Alanine | 2-3 | 0.07141135 | 0.009857701 | 0.132965005 | 0.015 |
| PAAP | Alanine | 2-4 | 0.09185910 | 0.021302289 | 0.162415911 | 0.005 |
| PAAP | Arginine | 1-3 | 0.16386824 | 0.087498712 | 0.240237769 | <0.001 |
| PAAP | Arginine | 1-4 | 0.26999777 | 0.179294691 | 0.360700845 | <0.001 |
| PAAP | Arginine | 2-3 | 0.10894947 | 0.036036471 | 0.181862475 | <0.001 |
| PAAP | Arginine | 2-4 | 0.21507900 | 0.131501361 | 0.298656640 | <0.001 |
| PAAP | Arginine | 3-4 | 0.10612953 | 0.029107657 | 0.183151398 | 0.002 |
| PAAP | Aspartic_Acid | 1-2 | 0.25651331 | 0.138338089 | 0.374688532 | <0.001 |
| PAAP | Aspartic_Acid | 1-3 | 0.55334740 | 0.428806696 | 0.677888104 | <0.001 |
| PAAP | Aspartic_Acid | 1-4 | 0.99745246 | 0.849537111 | 1.145367800 | <0.001 |
| PAAP | Aspartic_Acid | 2-3 | 0.29683409 | 0.177930166 | 0.415738013 | <0.001 |
| PAAP | Aspartic_Acid | 2-4 | 0.74093915 | 0.604643711 | 0.877234579 | <0.001 |
| PAAP | Aspartic_Acid | 3-4 | 0.44410506 | 0.318500536 | 0.569709576 | <0.001 |
| PAAP | Carbohydrate | 1-2 | -12.39373862 | -16.486687649 | -8.300789583 | <0.001 |
| PAAP | Carbohydrate | 1-3 | -25.21001100 | -29.523425827 | -20.896596182 | <0.001 |
| PAAP | Carbohydrate | 1-4 | -46.50018167 | -51.623167334 | -41.377195997 | <0.001 |
| PAAP | Carbohydrate | 2-3 | -12.81627239 | -16.934459712 | -8.698085064 | <0.001 |
| PAAP | Carbohydrate | 2-4 | -34.10644305 | -38.826978023 | -29.385908076 | <0.001 |
| PAAP | Carbohydrate | 3-4 | -21.29017066 | -25.640430327 | -16.939910996 | <0.001 |
| PAAP | Cysteine | 1-2 | -0.14323827 | -0.161195556 | -0.125280993 | <0.001 |
| PAAP | Cysteine | 1-3 | -0.22442443 | -0.243348978 | -0.205499885 | <0.001 |
| PAAP | Cysteine | 1-4 | -0.43402588 | -0.456502312 | -0.411549445 | <0.001 |
| PAAP | Cysteine | 2-3 | -0.08118616 | -0.099254168 | -0.063118146 | <0.001 |
| PAAP | Cysteine | 2-4 | -0.29078760 | -0.311498337 | -0.270076871 | <0.001 |
| PAAP | Cysteine | 3-4 | -0.20960145 | -0.228687645 | -0.190515248 | <0.001 |
| PAAP | Energy | 1-2 | -277.35459530 | -344.845246861 | -209.863943733 | <0.001 |
| PAAP | Energy | 1-3 | -538.81673506 | -607.557723196 | -470.075746926 | <0.001 |
| PAAP | Energy | 1-4 | -1,124.04527308 | -1,196.675756822 | -1,051.414789335 | <0.001 |
| PAAP | Energy | 2-3 | -261.46213976 | -329.482925965 | -193.441353564 | <0.001 |
| PAAP | Energy | 2-4 | -846.69067778 | -917.812751886 | -775.568603678 | <0.001 |
| PAAP | Energy | 3-4 | -585.22853802 | -654.000377599 | -516.456698436 | <0.001 |
| PAAP | Folate_total | 1-2 | 39.93202402 | 31.177643066 | 48.686404980 | <0.001 |
| PAAP | Folate_total | 1-3 | 56.13557428 | 46.909640540 | 65.361508020 | <0.001 |
| PAAP | Folate_total | 1-4 | 94.93124342 | 83.973724079 | 105.888762754 | <0.001 |
| PAAP | Folate_total | 2-3 | 16.20355026 | 7.395187291 | 25.011913223 | <0.001 |
| PAAP | Folate_total | 2-4 | 54.99921939 | 44.902499120 | 65.095939666 | <0.001 |
| PAAP | Folate_total | 3-4 | 38.79566914 | 29.490928211 | 48.100410061 | <0.001 |
| PAAP | Glutamic_Acid | 1-2 | -2.35590878 | -2.610137031 | -2.101680523 | <0.001 |
| PAAP | Glutamic_Acid | 1-3 | -3.78783667 | -4.055758876 | -3.519914471 | <0.001 |
| PAAP | Glutamic_Acid | 1-4 | -7.21829811 | -7.536505767 | -6.900090463 | <0.001 |
| PAAP | Glutamic_Acid | 2-3 | -1.43192790 | -1.687723795 | -1.176131999 | <0.001 |
| PAAP | Glutamic_Acid | 2-4 | -4.86238934 | -5.155599284 | -4.569179392 | <0.001 |
| PAAP | Glutamic_Acid | 3-4 | -3.43046144 | -3.700672214 | -3.160250669 | <0.001 |
| PAAP | Glycine | 1-2 | -0.07697598 | -0.129124081 | -0.024827880 | <0.001 |
| PAAP | Glycine | 1-3 | -0.06957783 | -0.124534882 | -0.014620788 | 0.006 |
| PAAP | Glycine | 1-4 | -0.16906440 | -0.234336157 | -0.103792644 | <0.001 |
| PAAP | Glycine | 2-4 | -0.09208842 | -0.152232569 | -0.031944272 | <0.001 |
| PAAP | Glycine | 3-4 | -0.09948657 | -0.154913051 | -0.044060081 | <0.001 |
| PAAP | Histidine | 1-2 | -0.04446286 | -0.078598519 | -0.010327193 | 0.005 |
| PAAP | Histidine | 1-3 | -0.03716355 | -0.073137927 | -0.001189182 | 0.040 |
| PAAP | Histidine | 1-4 | -0.08595660 | -0.128682889 | -0.043230317 | <0.001 |
| PAAP | Histidine | 2-4 | -0.04149375 | -0.080863548 | -0.002123945 | 0.034 |
| PAAP | Histidine | 3-4 | -0.04879305 | -0.085074712 | -0.012511385 | 0.003 |
| PAAP | Isoleucine | 1-2 | -0.10700239 | -0.165230166 | -0.048774616 | <0.001 |
| PAAP | Isoleucine | 1-3 | -0.12099121 | -0.182355415 | -0.059627013 | <0.001 |
| PAAP | Isoleucine | 1-4 | -0.24972616 | -0.322607606 | -0.176844708 | <0.001 |
| PAAP | Isoleucine | 2-4 | -0.14272377 | -0.209879806 | -0.075567727 | <0.001 |
| PAAP | Isoleucine | 3-4 | -0.12873494 | -0.190623313 | -0.066846574 | <0.001 |
| PAAP | Leucine | 1-2 | -0.24393380 | -0.340368830 | -0.147498772 | <0.001 |
| PAAP | Leucine | 1-3 | -0.31672433 | -0.418353813 | -0.215094852 | <0.001 |
| PAAP | Leucine | 1-4 | -0.62265666 | -0.743360650 | -0.501952673 | <0.001 |
| PAAP | Leucine | 2-4 | -0.37872286 | -0.489944604 | -0.267501117 | <0.001 |
| PAAP | Leucine | 3-4 | -0.30593233 | -0.408429920 | -0.203434737 | <0.001 |
| PAAP | Lysine | 1-2 | 0.21290935 | 0.115948677 | 0.309870027 | <0.001 |
| PAAP | Lysine | 1-3 | 0.46226408 | 0.360080637 | 0.564447518 | <0.001 |
| PAAP | Lysine | 1-4 | 0.83504846 | 0.713686545 | 0.956410384 | <0.001 |
| PAAP | Lysine | 2-3 | 0.24935473 | 0.151796163 | 0.346913288 | <0.001 |
| PAAP | Lysine | 2-4 | 0.62213911 | 0.510311123 | 0.733967101 | <0.001 |
| PAAP | Lysine | 3-4 | 0.37278439 | 0.269728103 | 0.475840670 | <0.001 |
| PAAP | Methionine | 1-2 | -0.03778364 | -0.067524241 | -0.008043039 | 0.006 |
| PAAP | Methionine | 1-3 | -0.03280260 | -0.064145169 | -0.001460026 | 0.036 |
| PAAP | Methionine | 1-4 | -0.07752392 | -0.114749078 | -0.040298763 | <0.001 |
| PAAP | Methionine | 2-4 | -0.03974028 | -0.074041110 | -0.005439451 | 0.015 |
| PAAP | Methionine | 3-4 | -0.04472132 | -0.076331620 | -0.013111026 | 0.002 |
| PAAP | Niacin | 1-2 | 0.86293220 | 0.507968927 | 1.217895478 | <0.001 |
| PAAP | Niacin | 1-3 | 1.51289550 | 1.138812208 | 1.886978795 | <0.001 |
| PAAP | Niacin | 1-4 | 2.06760436 | 1.623310591 | 2.511898139 | <0.001 |
| PAAP | Niacin | 2-3 | 0.64996330 | 0.292811219 | 1.007115380 | <0.001 |
| PAAP | Niacin | 2-4 | 1.20467216 | 0.795281148 | 1.614063177 | <0.001 |
| PAAP | Niacin | 3-4 | 0.55470886 | 0.177430180 | 0.931987546 | <0.001 |
| PAAP | Pantothenic_Acid | 1-2 | 0.36460487 | 0.262495558 | 0.466714188 | <0.001 |
| PAAP | Pantothenic_Acid | 1-3 | 0.61904347 | 0.511434060 | 0.726652882 | <0.001 |
| PAAP | Pantothenic_Acid | 1-4 | 1.11911375 | 0.991307476 | 1.246920020 | <0.001 |
| PAAP | Pantothenic_Acid | 2-3 | 0.25443860 | 0.151699648 | 0.357177549 | <0.001 |
| PAAP | Pantothenic_Acid | 2-4 | 0.75450888 | 0.636742788 | 0.872274963 | <0.001 |
| PAAP | Pantothenic_Acid | 3-4 | 0.50007028 | 0.391541675 | 0.608598879 | <0.001 |
| PAAP | Phenylalanine | 1-2 | -0.23412672 | -0.288605274 | -0.179648156 | <0.001 |
| PAAP | Phenylalanine | 1-3 | -0.34690705 | -0.404320082 | -0.289494013 | <0.001 |
| PAAP | Phenylalanine | 1-4 | -0.66928551 | -0.737474207 | -0.601096806 | <0.001 |
| PAAP | Phenylalanine | 2-3 | -0.11278033 | -0.167594821 | -0.057965843 | <0.001 |
| PAAP | Phenylalanine | 2-4 | -0.43515879 | -0.497990735 | -0.372326848 | <0.001 |
| PAAP | Phenylalanine | 3-4 | -0.32237846 | -0.380281911 | -0.264475007 | <0.001 |
| PAAP | Proline | 1-2 | -0.80114622 | -0.887545422 | -0.714747025 | <0.001 |
| PAAP | Proline | 1-3 | -1.31431772 | -1.405370791 | -1.223264647 | <0.001 |
| PAAP | Proline | 1-4 | -2.47565877 | -2.583801300 | -2.367516244 | <0.001 |
| PAAP | Proline | 2-3 | -0.51317150 | -0.600103456 | -0.426239535 | <0.001 |
| PAAP | Proline | 2-4 | -1.67451255 | -1.774159632 | -1.574865464 | <0.001 |
| PAAP | Proline | 3-4 | -1.16134105 | -1.253171893 | -1.069510213 | <0.001 |
| PAAP | Riboflavin | 1-2 | 0.16973397 | 0.127604288 | 0.211863656 | <0.001 |
| PAAP | Riboflavin | 1-3 | 0.29584129 | 0.251442296 | 0.340240275 | <0.001 |
| PAAP | Riboflavin | 1-4 | 0.52237244 | 0.469640343 | 0.575104527 | <0.001 |
| PAAP | Riboflavin | 2-3 | 0.12610731 | 0.083717846 | 0.168496781 | <0.001 |
| PAAP | Riboflavin | 2-4 | 0.35263846 | 0.304048891 | 0.401228036 | <0.001 |
| PAAP | Riboflavin | 3-4 | 0.22653115 | 0.181752908 | 0.271309392 | <0.001 |
| PAAP | Serine | 1-2 | -0.21493252 | -0.271792498 | -0.158072548 | <0.001 |
| PAAP | Serine | 1-3 | -0.31569051 | -0.375613238 | -0.255767787 | <0.001 |
| PAAP | Serine | 1-4 | -0.60185399 | -0.673023414 | -0.530684560 | <0.001 |
| PAAP | Serine | 2-3 | -0.10075799 | -0.157968580 | -0.043547400 | <0.001 |
| PAAP | Serine | 2-4 | -0.38692146 | -0.452499975 | -0.321342954 | <0.001 |
| PAAP | Serine | 3-4 | -0.28616347 | -0.346598055 | -0.225728894 | <0.001 |
| PAAP | Thiamin | 1-2 | -0.06834205 | -0.095789114 | -0.040894993 | <0.001 |
| PAAP | Thiamin | 1-3 | -0.13751199 | -0.166437480 | -0.108586500 | <0.001 |
| PAAP | Thiamin | 1-4 | -0.29824112 | -0.332595543 | -0.263886701 | <0.001 |
| PAAP | Thiamin | 2-3 | -0.06916994 | -0.096786243 | -0.041553630 | <0.001 |
| PAAP | Thiamin | 2-4 | -0.22989907 | -0.261554681 | -0.198243456 | <0.001 |
| PAAP | Thiamin | 3-4 | -0.16072913 | -0.189901701 | -0.131556563 | <0.001 |
| PAAP | Total_lipid_fat | 1-2 | 7.78063882 | 6.101182108 | 9.460095541 | <0.001 |
| PAAP | Total_lipid_fat | 1-3 | 14.60520145 | 12.835281172 | 16.375121730 | <0.001 |
| PAAP | Total_lipid_fat | 1-4 | 27.96929453 | 25.867183653 | 30.071405411 | <0.001 |
| PAAP | Total_lipid_fat | 2-3 | 6.82456263 | 5.134749901 | 8.514375352 | <0.001 |
| PAAP | Total_lipid_fat | 2-4 | 20.18865571 | 18.251682122 | 22.125629294 | <0.001 |
| PAAP | Total_lipid_fat | 3-4 | 13.36409308 | 11.579054285 | 15.149131877 | <0.001 |
| PAAP | Tryptophan | 1-2 | -0.04955289 | -0.063338479 | -0.035767302 | <0.001 |
| PAAP | Tryptophan | 1-3 | -0.06810074 | -0.082628887 | -0.053572596 | <0.001 |
| PAAP | Tryptophan | 1-4 | -0.13251954 | -0.149774431 | -0.115264658 | <0.001 |
| PAAP | Tryptophan | 2-3 | -0.01854785 | -0.032418445 | -0.004677257 | 0.003 |
| PAAP | Tryptophan | 2-4 | -0.08296665 | -0.098866034 | -0.067067274 | <0.001 |
| PAAP | Tryptophan | 3-4 | -0.06441880 | -0.079071047 | -0.049766559 | <0.001 |
| PAAP | Tyrosine | 1-2 | -0.08377686 | -0.127597564 | -0.039956152 | <0.001 |
| PAAP | Tyrosine | 1-3 | -0.09843135 | -0.144612450 | -0.052250253 | <0.001 |
| PAAP | Tyrosine | 1-4 | -0.19448548 | -0.249334161 | -0.139636802 | <0.001 |
| PAAP | Tyrosine | 2-4 | -0.11070862 | -0.161248509 | -0.060168738 | <0.001 |
| PAAP | Tyrosine | 3-4 | -0.09605413 | -0.142629704 | -0.049478556 | <0.001 |
| PAAP | Valine | 1-2 | -0.11049296 | -0.176742278 | -0.044243639 | <0.001 |
| PAAP | Valine | 1-3 | -0.12463900 | -0.194456822 | -0.054821173 | <0.001 |
| PAAP | Valine | 1-4 | -0.25090952 | -0.333831224 | -0.167987813 | <0.001 |
| PAAP | Valine | 2-4 | -0.14041656 | -0.216824116 | -0.064009005 | <0.001 |
| PAAP | Valine | 3-4 | -0.12627052 | -0.196684724 | -0.055856319 | <0.001 |
| PAAP | VitaminA_mcg | 1-2 | 145.06333225 | 100.575071248 | 189.551593245 | <0.001 |
| PAAP | VitaminA_mcg | 1-3 | 276.39665766 | 229.512046377 | 323.281268939 | <0.001 |
| PAAP | VitaminA_mcg | 1-4 | 482.09218263 | 426.407950634 | 537.776414623 | <0.001 |
| PAAP | VitaminA_mcg | 2-3 | 131.33332541 | 86.570737112 | 176.095913712 | <0.001 |
| PAAP | VitaminA_mcg | 2-4 | 337.02885038 | 285.719051445 | 388.338649319 | <0.001 |
| PAAP | VitaminA_mcg | 3-4 | 205.69552497 | 158.410429106 | 252.980620836 | <0.001 |
| PAAP | VitaminB12 | 1-2 | 1.05254329 | 0.463561605 | 1.641524969 | <0.001 |
| PAAP | VitaminB12 | 1-3 | 2.46037247 | 1.839665420 | 3.081079518 | <0.001 |
| PAAP | VitaminB12 | 1-4 | 4.27093971 | 3.533734171 | 5.008145257 | <0.001 |
| PAAP | VitaminB12 | 2-3 | 1.40782918 | 0.815215671 | 2.000442692 | <0.001 |
| PAAP | VitaminB12 | 2-4 | 3.21839643 | 2.539104160 | 3.897688694 | <0.001 |
| PAAP | VitaminB12 | 3-4 | 1.81056725 | 1.184558167 | 2.436576325 | <0.001 |
| PAAP | VitaminB6 | 1-2 | 0.82512325 | 0.228406065 | 1.421840437 | 0.002 |
| PAAP | VitaminB6 | 1-3 | 1.95422680 | 1.325367574 | 2.583086022 | <0.001 |
| PAAP | VitaminB6 | 1-4 | 3.26639343 | 2.519505656 | 4.013281203 | <0.001 |
| PAAP | VitaminB6 | 2-3 | 1.12910355 | 0.528706833 | 1.729500261 | <0.001 |
| PAAP | VitaminB6 | 2-4 | 2.44127018 | 1.753056296 | 3.129484061 | <0.001 |
| PAAP | VitaminB6 | 3-4 | 1.31216663 | 0.677935742 | 1.946397521 | <0.001 |
| PAAP | VitaminC_total_ascorbic_acid | 1-2 | 35.75383839 | 28.126161964 | 43.381514817 | <0.001 |
| PAAP | VitaminC_total_ascorbic_acid | 1-3 | 54.37158970 | 46.333050178 | 62.410129220 | <0.001 |
| PAAP | VitaminC_total_ascorbic_acid | 1-4 | 96.88007171 | 87.332804765 | 106.427338657 | <0.001 |
| PAAP | VitaminC_total_ascorbic_acid | 2-3 | 18.61775131 | 10.943040454 | 26.292462163 | <0.001 |
| PAAP | VitaminC_total_ascorbic_acid | 2-4 | 61.12623332 | 52.328979056 | 69.923487586 | <0.001 |
| PAAP | VitaminC_total_ascorbic_acid | 3-4 | 42.50848201 | 34.401277933 | 50.615686092 | <0.001 |
| PAAP | VitaminD_IU | 1-2 | 5.17375455 | 2.470985673 | 7.876523431 | <0.001 |
| PAAP | VitaminD_IU | 1-3 | 11.05037433 | 8.202021405 | 13.898727253 | <0.001 |
| PAAP | VitaminD_IU | 1-4 | 18.50068158 | 15.117730533 | 21.883632634 | <0.001 |
| PAAP | VitaminD_IU | 2-3 | 5.87661978 | 3.157184854 | 8.596054699 | <0.001 |
| PAAP | VitaminD_IU | 2-4 | 13.32692703 | 10.209733312 | 16.444120750 | <0.001 |
| PAAP | VitaminD_IU | 3-4 | 7.45030725 | 4.577623929 | 10.322990580 | <0.001 |
| PAAP | VitaminE_alpha_tocopheraol | 1-2 | 1.33689115 | 1.072912934 | 1.600869370 | <0.001 |
| PAAP | VitaminE_alpha_tocopheraol | 1-3 | 2.24498681 | 1.966789461 | 2.523184154 | <0.001 |
| PAAP | VitaminE_alpha_tocopheraol | 1-4 | 4.02063530 | 3.690223997 | 4.351046606 | <0.001 |
| PAAP | VitaminE_alpha_tocopheraol | 2-3 | 0.90809566 | 0.642489672 | 1.173701639 | <0.001 |
| PAAP | VitaminE_alpha_tocopheraol | 2-4 | 2.68374415 | 2.379289243 | 2.988199055 | <0.001 |
| PAAP | VitaminE_alpha_tocopheraol | 3-4 | 1.77564849 | 1.495074808 | 2.056222179 | <0.001 |
| PAAP | VitaminK | 1-2 | 36.16131083 | 25.597908214 | 46.724713447 | <0.001 |
| PAAP | VitaminK | 1-3 | 50.08814677 | 38.955748817 | 61.220544714 | <0.001 |
| PAAP | VitaminK | 1-4 | 87.84756349 | 74.625761857 | 101.069365124 | <0.001 |
| PAAP | VitaminK | 2-3 | 13.92683593 | 3.298296366 | 24.555375503 | 0.004 |
| PAAP | VitaminK | 2-4 | 51.68625266 | 39.503127220 | 63.869378099 | <0.001 |
| PAAP | VitaminK | 3-4 | 37.75941672 | 26.531926727 | 48.986906722 | <0.001 |
| PAAP | protein | 1-4 | -1.82386963 | -3.319677987 | -0.328061275 | 0.009 |
| PAAP | protein | 3-4 | -1.66282128 | -2.933009245 | -0.392633307 | 0.004 |
